# Sustainable wet-spun cellulose-*Moringa oleifera* composite fibres for potential water purification†

**DOI:** 10.1039/d5ra02386f

**Published:** 2025-05-28

**Authors:** Abimbola Oluwatayo Orisawayi, Prithivi Boylla, Krzysztof K. Koziol, Sameer S. Rahatekar

**Affiliations:** a Composites and Advanced Materials Centre, Faculty of Engineering and Applied Sciences, Cranfield University College Road, Cranfield Bedfordshire MK43 0AL UK abimbola.orisawayi@cranfield.ac.uk bimboris_t@yahoo.com S.S.Rahatekar@cranfield.ac.uk; b Department of Mechanical Engineering, School of Engineering and Engineering Technology, Olusegun Agagu University of Science and Technology (OAUSTECH) Okitipupa Nigeria ao.orisawayi@oaustech.edu.ng

## Abstract

This study explores a pioneering fabrication of novel cellulose-*Moringa oleifera* (*M. oleifera*) composite fibres (CeL-MoFs) and comparable pure regenerated cellulose fibres (CeFs) using the ionic liquid 1-ethyl-3-methylimidazolium diethyl phosphate (EMIM DEP) and the simple traditional wet-spinning process. The composites, CeL-MoFs at 0.5%, 1%, 2%, and 3%, were characterised. Fourier-transform infrared (FTIR) spectroscopy and scanning electron microscopy with energy-dispersive X-ray spectroscopy (SEM-EDX) confirmed the successful integration of *M. oleifera* seed powder (MoP) into the cellulose matrix. The results of preliminary adsorption studies demonstrated high selectivity for copper ions (Cu^2+^), with no detectable selectivity towards nickel (Ni^2+^) or cadmium (Cd^2+^). Thermogravimetric analysis (TGA) and derivative thermogravimetric (DTG) analysis revealed thermal stability variations with increasing MoP content, while atomic force microscopy (AFM) showed surface roughness and fibre defects. Rheological testing validated spinnability, and tensile analysis identified CeL-MoFs (2%) as the optimal composite, balancing mechanical strength and adsorption efficiency. These novel CeL-MoF composites, fabricated using EMIM DEP, are proposed as scalable, eco-friendly materials for selective heavy metal removal. Future work will focus on adsorption kinetics, thermodynamic modelling, and scaling production for industrial water purification applications.

## Abstract 2

This study explores a pioneering fabrication of novel cellulose-*Moringa oleifera* (CeL-MoFs) composite fibres using the ionic liquid 1-ethyl-3-methylimidazolium diethyl phosphate (EMIM DEP) through a simple traditional wet-spinning process.

## Graphical abstract

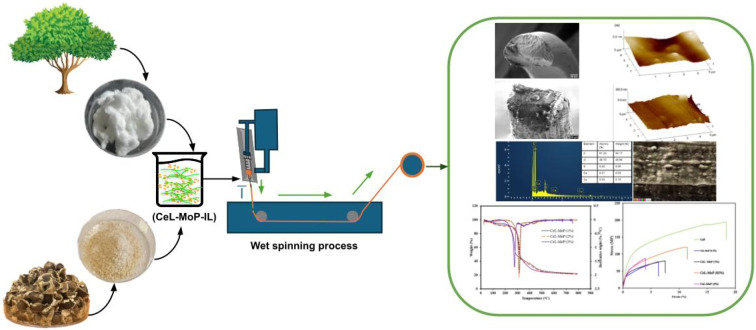

## 1. Introduction

In recent days, the rapid growth in global industrialisation aimed at improving human life has significantly affected the environment.^1,2^ Studies have emphasised the widespread environmental effects of environmental pollution, which adversely affect land, air, and water.^3,4^ Among these, water pollution has emerged as a pressing concern, posing severe risks to human health and contributing to numerous hazardous health-related issues.^5,6^ Water pollution is majorly caused by pollutant discharge into water bodies and contaminants such as pathogens, heavy metals, dyes, and pigments from industrial or domestic waste, which may either be absorbed by the soil or flow directly into the water system, affecting the concentration of underground and surface water.^7,8^

Among these pollutants, heavy metal ions, such as chromium (Cr), lead (Pb), cadmium (Cd), mercury (Hg), copper (Cu), nickel (Ni), and manganese (Mn), stand out for their toxic and persistent nature, contributing to significant risks to ecosystems and human health.^5,9,10^ Heavy metals primarily originate from industrial activities such as machinery manufacturing, mineral smelting, electroplating, electronics production, oil refining, and chemical processing.^9,11^ Therefore, heavy metal ion contamination in water bodies is a critical problem. Developing effective methods for removing these toxic metals remains a prominent focus in environmental research. Several conventional and advanced technologies have been used to address water challenges, including traditional water treatment methods, such as oxidation, electro-precipitation, membrane separation, coagulation–flocculation, evaporation, flotation, and ion exchange, but these methods are often inadequate in addressing efficient water treatment^1,12^

In heavy metal ion removal, an adsorption method is usually considered one of the effective physical methods preferred for addressing the removal of metal ions from an aqueous solution because of its advantages of multiple adsorbent selection, high-efficiency removal, selective compliance, simple and easy operation, good reversibility and low cost.^13^ There is a demand for efficient, sustainable materials as an alternative to existing adsorbent materials, sorbent biopolymers and biomaterials.^14^

Studies on the application of novel chemically modified cellulose for the adsorption of heavy metal ions by Fakhre and Ibrahim.^15^ revealed that some effective chelating ion-exchange materials are composed of biopolymers and their derivatives, which is attributed to the presence of various functional groups, such as –NH_2_ and –OH, which readily interact to form bonds with other chemical entities, which may include metal ions from aqueous solution.^16,17^ These biopolymers may comprise cellulose, alginates, proteins, chitin, and chitin derivatives, such as chitosan, to exhibit outstanding efficiency in reducing metal ion concentrations to considerable concentrations;^5,13,18^ further reports from these studies stated that cellulose and most of its derivatives can adsorb metals effectively using their hydroxyl groups, which can be replaced by other functional groups.^19–22^

Modified cellulose can exhibit a 40–80% higher adsorption capacity for heavy metal ions than unmodified cellulose.^23,24^ Therefore, from the sustainability perspective, biopolymers, such as cellulose, are used in various applications, such as medical devices, construction applications, textiles, pharmaceuticals, aerospace, and automotive, including water treatment.^10^ Cellulose is the most abundant natural polymer produced by plants and may be biodegradable. It has been discussed for ages as one of the oldest polymeric materials known in the chemical industries; cellulose remains a significant focus and important polymer in research to date, and it has attracted wide interest even in polymer science owing to its excellent biocompatibility, strong and structural forming ability, and environmentally sustainable properties, making it a key material for advanced applications.^25,26^

Cellulose polymer consists of β(1→4)-polysaccharide with an extensive network of intra- and inter-hydrogen bonds, which enables it to adopt a highly ordered structure.^23,27^ This is responsible for cellulose having desirable chemical and mechanical properties for healing, bactericide and fungicide, drug delivery, and adsorbents for organic and inorganic pollutants.^27^*M. oleifera* seed powder has been extensively studied owing to its bioactive properties and excellent adsorption capabilities for heavy metals and other contaminants. The seed's high protein content provides functional groups, such as amino acids and carboxyl, enabling interactions with pollutants through various adsorption mechanisms.^28–30^

*M. oleifera* seeds also possess antimicrobial properties, making them suitable for water purification applications. However, their direct use in filtration systems is limited by their mechanical instability and tendency to disperse in aqueous environments, as previous studies have shown that it can cause secondary pollution if not properly encapsulated.^10^ To address these limitations, we propose using *M. oleifera* seed powder and polymers, such as cellulose, which could provide a synergistic approach. Cellulose, being a mechanically robust and biodegradable polymer, offers a stable structural matrix that can encapsulate and support the active components of *M. oleifera*.

Previous studies have explored cellulose's potential, including using ionic liquids and *M. oleifera* separately for water purification.^31–33^ Cellulose-based materials have been extensively investigated owing to their mechanical strength, chemical stability, and ability to adsorb heavy metals; *M. oleifera* seed powder is valued for its bioactive properties and superior adsorption capabilities.^34,35^ However, the direct use of *M. oleifera* is hindered by mechanical instability and dispersion issues in aqueous environments. Similarly, although biopolymer composites, such as alginate-*M. oleifera*,^10^ have been developed to address these limitations, combining cellulose and *M. oleifera* using green, scalable techniques, such as ionic liquid-based wet spinning, remains unexplored. This gap must be addressed by highlighting the need for innovative, sustainable solutions.

To prepare cellulose solutions, ionic liquids (IL) have been widely used because they offer a promising alternative to the commonly used acidic solvents in dissolving cellulose. Ionic liquids (ILs), with melting points below 100 °C, have garnered attention owing to their exceptional properties, including high thermal stability, non-flammability, low vapour pressure, and remarkable polymer solubility.^36^ Carefully selected ionic liquids with low toxicity and excellent recyclability offer significant environmental and safety advantages over traditional fibre production processes. The ionic liquid used in the study is imidazolium-based, such as 1-ethyl-3-methylimidazolium diethyl phosphate, which was reported in one study as an eco-friendly approach toward downstream processing of bacterial biomass for the extraction of an intracellular potential bioplastic material, polyhydroxyalkanoates, replacing chlorinated organic solvents.^37^ ILs are frequently used for dissolution with a sustainable and efficient medium for processing materials, preserving their bioactive properties to enable uniform dispersion and enhancing their integration with other materials like cellulose.^38^

The novelty of this study lies in the pioneering integration of *M. oleifera* (MoP) with cellulose (CeL) using ionic liquids through wet spinning, a fibre production technique in which a polymer solution is extruded through a spinneret into a coagulation bath (in this case, water was used as a coagulation bath because it is eco-friendly). Wet-spinning is particularly advantageous for processing materials that require precise control over fibre morphology and properties, making it ideal for creating biopolymer composites with enhanced mechanical stability and functional performance. By leveraging cellulose's exceptional mechanical strength and MoP's superior adsorption capabilities, this study aims to develop robust composite fibres tailored for water treatment applications. In addition, we expect that this pioneering CeL-MoF composite would deliver a sustainable and scalable solution for water purification, merging MoP's superior adsorption capabilities with cellulose's mechanical durability. MoP may affect mechanical properties, but it could still be suitable for application in water treatment. This advancement has the potential to be transformative for industrial-scale water treatment and environmental remediation technologies.

In this work, we first developed pure CeF fibres and their composites (CeL-MoF composite fibres) through an ionic liquid-mediated wet-spinning process using the same wet spinning setup as our previous studies.^10^ Specific characterisation of the composite fibres was performed using FTIR to analyse chemical interactions and bonding, TGA to evaluate thermal stability, and SEM-EDX to study morphological features and elemental composition, with further characterisation using AFM to assess the surface topography and rheology to examine the flow and viscoelastic properties of the spinning solution. In addition, the mechanical properties of the composite fibres were assessed to ensure their robustness and suitability for practical applications. Tensile testing was conducted to determine tensile strength, Young's modulus, and elongation at break, providing insights into the ability of the fibres to withstand mechanical stresses. The composite developed demonstrated structural and functional advantages, such as an eco-friendly, efficient, and sustainable solution, for potential water purification applications.

## 2. Materials and methods

### 2.1 Materials

The cellulose used in this study was high-purity cellulose. Pulp sheets were procured from Rayonier, Fernandina Beach (USA), with a degree of polymerization (DP) of 890. The Ionic Liquid (IL) used as a solvent was 1-ethyl-3-methylimidazolium diethyl phosphate (C_10_H_21_N_2_O_4_P), EMIM DEP, molecular weight (*M*_w_):264.26 g mol^−1^, ≤ 100% (specifically 99% high purity), product number: 671541, CAS-No.: 848641-69-0, CAS-No.: 848641-69-0, procured from Sigma-Aldrich, UK. *M. oleifera* seeds used were the ones previously procured through our trusted supplier (Purely Agro Ltd, “DGT”d 2, London, UK).^10,34^ Other chemicals used for preliminary adsorption studies include copper(ii) acetate monohydrate (C_4_H_6_CuO_4_·H_2_O), cadmium acetate dihydrate (C_4_H_6_CdO_4_·2H_2_O), and nickel(ii) acetate tetrahydrate (C_4_H_6_NiO_4_·2H_2_O) procured from Sigma-Aldrich, UK. All chemicals were of high purity, and deionized water was supplied by the university laboratory supplier, as reported in previous studies.^10^

### 2.2 Methods

#### 2.2.1 Preparation of CeL–IL, MoP–IL, and CeL–MoP–IL solutions

The cellulose sheet was first blended into fine fragments using a clean blender (KENWOOD Easy chopper equipped with quad blade system technology mini chopper CH61.100WH, China). This initial step is important for increasing the surface area, facilitating the efficient dissolution of the cellulose fragments in the IL (ionic liquid) during dissolution. A 6 wt% cellulose solution (CeL–IL) was prepared by dissolving 6 g of finely blended cellulose in 94 g of ionic liquid (IL). The solution was maintained at 80 °C and stirred continuously using a mechanical stirrer at 200 rpm for 6 hours to ensure complete dissolution. After dissolution, the cellulose solution was cooled to 60 °C. Separately, a 10 wt% solution of *M. oleifera* seed powder (MoP–IL) was prepared by dispersing the seed powder (MoP) of 10 g in 90 g of IL. The MoP–IL solution was also stirred continuously at 200 rpm at 60 °C. The MoP–IL solution was allowed to stand for 24 hours to ensure uniform dispersion and stabilization of the *M. oleifera* seed particles in the IL. Subsequently, the MoP–IL solution was stirred for another 4 hours at 60 °C to improve homogeneity. The cooled CeL–IL solution was then mixed with the prepared CeL–MoP–IL solution at predetermined weight-by-weight (w/w) ratios of 95 : 5, 90 : 10, 80 : 20, and 70 : 30, These ratios were carefully selected to prevent agglomeration, which was observed during preliminary preparations at higher MoP concentrations. The resulting mixtures were stirred for an additional 4 hours. The final mixtures had cellulose concentrations of 5.7 wt%, 5.4 wt%, 4.8 wt%, and 4.2 wt%, respectively, and MoP concentrations of 0.5 wt%, 1.0 wt%, 2.0 wt%, and 3.0 wt%. The control solution consisting of 6 wt% CeL in IL served as a baseline for comparison. Each solution was loaded into a 60 mL syringe. To remove air bubbles and ensure uniformity, the samples were degassed using a vacuum oven. Table 1 presents a comprehensive summary of the prepared samples, serving as a reference point for the subsequent characterisation and evaluation of their properties. These compositions were linked to the preparation of solutions used in the wet spinning process to produce fibres.

**Table 1:** Composition of CeL and MoP solutions in ionic liquid for fiber production

Sample name	CeL (wt%)	MoP (wt%)	IL (wt%)	Description
CeL–IL	6.0	0.0	94.0	Control with only cellulose in IL
MoP–IL	0.0	10.0	90.0	MoP solution in IL
CeL–MoP–IL (0.5%)	5.7	0.5	93.8	95 : 5 (w/w) mix of CeL–IL and MoP–IL
CeL–MoP–IL (1%)	5.4	1.0	93.6	90 : 10 (w/w) mix of CeL–IL and MoP–IL
CeL–MoP–IL (2%)	4.8	2.0	93.2	80 : 20 (w/w) mix of CeL–IL and MoP–IL
CeL–MoP–IL (3%)	4.2	3.0	92.8	70 : 30 (w/w) mix of CeL–IL and MoP–IL

#### 2.2.2 Wet spinning preparation of fibres

**Fig. 1:**
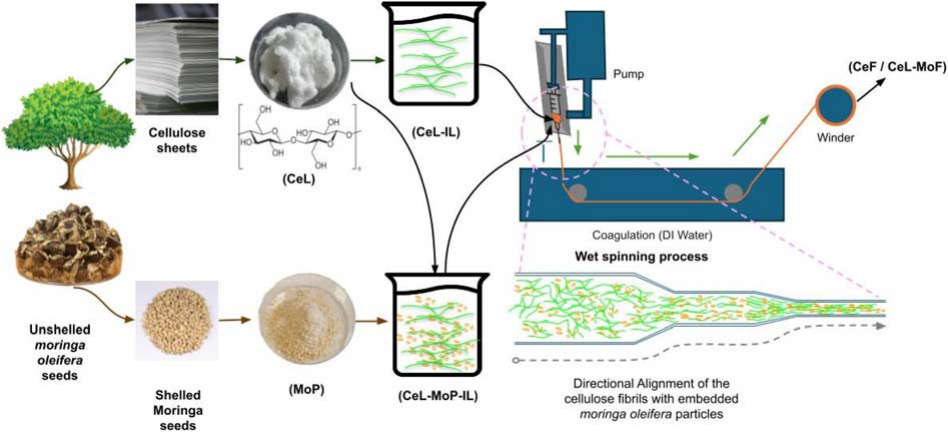
Schematic of the fibre filaments through the traditional wet spinning process.

The fabrication of the fibres was conducted in a lab-scale wet-spinning setup (Fig. 1). Each sample solution was extruded individually through a needle of 85 μm diameter on a single (Chemyx™) fusion syringe pump at a flow rate of 1 mL min^−1^ using approximately 2 litres of deionised water as a coagulation bath. The samples were processed sequentially to ensure consistency and accuracy in the fabrication process. The fabricated fibres were continuously collected on rollers mounted on a Filabot™ winder. Finally, the fabricated fibres were soaked in water and rinsed several times to remove traces of residual IL in the fibres.^39,40^ The fibres were dried in a clean open-air laboratory for several days before collection. Before characterisation, the samples were conditioned. This process was conducted in a Gallen Kamp TH 340 L/−40 °C environmental chamber regulated in the range of 21–25 °C and a relative humidity of 45% for a complete 24 hours.^41^

#### 2.2.3 Preparation of heavy metal ion solutions

We adopted this method in the present study to evaluate preliminary adsorption studies for the samples developed: pure CeF and CeL-MoF. The salts used for the experiments were selected based on their proven effectiveness in previous studies,^11^ as reported by Orisawayi *et al.*^10,34^ For the selective adsorption experiments, a combination of heavy metal ion solutions was prepared, containing copper (Cu^2+^), cadmium (Cd^2+^), and nickel (Ni^2+^) ions, each at a concentration of 50 mg L^−1^ in aqueous solutions. The resulting stock solution contained 50 mg L^−1^ of each heavy metal ion, Cu^2+^, Cd^2+^, and Ni^2+^. This solution was formulated by dissolving precise amounts of copper(ii) acetate, nickel(ii) acetate, and cadmium(ii) acetate salts in deionised water to achieve concentrations in a total volume of 100 mL.

### 2.3 Characterisations and instrumentations

#### 2.3.1 Rheology of CeL–IL and CeL–MoP–IL solutions

The rheological properties of freshly prepared CeL–IL and CeL–MoP–IL solutions were investigated before the solution was used to produce the fibre using a flow step. Each experiment was repeated on each sample three times to ensure precision and reliability. The measurements were conducted using a TA Instruments rheometer setup (AR-2000ex Rheometer 8F382@lab) equipped with a standard 40 mm diameter parallel plate (40SST Plate). Before the experiments, the instruments were calibrated, as established in previous studies.^34^ The only variation was the controlled temperature set to 65 ± 1 °C because of the nature of CeL–IL and CeL–MoP–IL solutions. In a rotational mapping procedure with three iterations, the upper plate was positioned at a 5 mm gap, and inertia was set at 3.681 μN m s^2^, with a shear rate ranging from 0.1 to 1000 s^−1^. Data were collected at 40 specific points. Particular attention was paid to shear rates of 0.1 s^−1^ to determine the zero-shear viscosity, as this value represents the Newtonian plateau of the sample.

#### 2.3.2 Fourier transform infrared (FT-IR) of the composites

The FTIR analysis of the fibres (CeF and CeL-MoF) of both the adsorbed and unabsorbed fibre samples was investigated using the Thermo Scientific™ Nicolet iS™ 10 FTIR Spectrometer from Verona, Madison, USA. The samples were prepared, and measurements were taken. For a spectral range over the wavelength of 4000–500 cm^−1^ and a resolution of 4 cm^−1^ at room temperature, a total of 50 scans were collected utilizing the attenuated total reflectance (ATR) technique with a Smart iTR accessory fitted with a diamond crystal. Omnic™ software was used for the initial analysis, and Microsoft Excel was used to further analyse the data.

#### 2.3.3 Thermogravimetric analysis (TGA) of CeF and CeL-MoF

The evaluation of the TGA of the samples (CeF and CeL-MoF) was evaluated using the TA Instrument Q500 Thermogravimetric Analyser (USA). Approximately 5 mg of the samples were used in a platinum crucible for characterisation.^42^ The samples were heated from room temperature to a final temperature of 800 °C at 20 °C min^−1^ under a nitrogen atmosphere with a flow rate of 40 mL min^−1^ and a purge flow of 60 mL min^−1^.

#### 2.3.4 SEM-EDX characterisation of material properties

The images of the individual fibres were analysed using a Scanning Electron Microscope (SEM) S8000 (TESCAN, Kohoutovice, Czech Republic). To obtain a high-resolution image and ensure accurate and reliable results from the morphologies of each of the samples, the samples were coated with an AU 10 nm (gold).^43^

#### 2.3.5 Atomic force microscope (AFM) of CeF and CeL-MoF

The Atomic Force Microscope (AFM), with typical high-resolution two-dimensional (2-D) and three-dimensional (3-D) resolution surface topographic imaging of the samples, was conducted using the AFM – Bruker Veeco (V) Dimension 3100 (Digital Instruments, Santa Barbara, California, USA). The AFM was equipped with a Nasoscope V dimension controller using nano-sensor tips PPP-NCHR Silicon. Samples were allowed to air-dry before analysis in tapping mode.^44,45^ A scan speed of 0.4000 Hz, 1024 lines, and 1024 samples/1024 samples per line, and a drive amplitude of 123.7 mV were used. These settings were used to achieve an optimised value with the highest resolution necessary to evaluate the detailed topography analysis properties of all the samples. Initial imaging was performed using Nasoscope V7.30r1sr3 software, and ImageJ software was subsequently used for post-processing and image modifications.

#### 2.3.6 Mechanical properties of CeF and CeL-MoF fibres

Tensile testing was conducted on samples of CeF and CeL-MoF to ensure high precision, enabling real-time observation of fibre deformation under high-resolution imaging. We used *in situ* mechanical testing on a TESCAN SEM Vega 3 Oxford Instruments system equipped with a Deben micro test tensile stage controller, operated using Deben Microtest software V6.3.4 equipped with a digital extensometer calibration (Edmunds, Suffolk, UK) testing stage. Optical Leica S9D was used to measure the fibre diameter before the test. The testing standard used is ASTM D3822, a standard method for evaluating the tensile properties of single filament fibres. Samples were cut to lengths of about 8 cm each and affixed onto tabs to prevent damage that may occur during testing.^10,46^ A constant speed rate of 1 mm min^−1^, with a uniform gauge length of about 10.2 mm, was used.

#### 2.3.7 SEM-energy dispersive X-ray spectroscopy (EDX) of CeF and CeL-MoF

The SEM-energy-dispersive X-ray spectroscopy (EDX) on the samples was performed on an S8000 model (TESCAN, Kohoutovice, Czech Republic) equipped with an Oxford Instruments ULTIM MAX 100 EDX system. Before the analysis, each sample was coated with an AU10 nm (gold) layer using a Quorum Q150T ES sputter coater (Quorum Technologies Ltd, UK). The analysis was conducted at an accelerating voltage of 20 kV and 1 nA beam current, and secondary electron imaging was used. The elemental composition and metal adsorption (Cu^2+^, Cd^2+^, and Ni^2+^) were analysed through spot analysis mode. EDX mapping with a resolution of 1024 pixels was used. Data processing was performed using Aztec version 6 software. Similar conditions were employed in previous studies to determine the elemental composition of fibers and quantify the adsorption of heavy metals (Cu^2+^, Cd^2+^, and Ni^2+^). The SEM-EDX methodology was adapted from our previous studies^10^ and has proven to be effective for analysing fibre morphology and elemental composition in hybrid composites.

## 3. Results and discussion

### 3.1 Rheology of freshly prepared CeL–IL and CeL–MoP–IL solutions

**Fig. 2:**
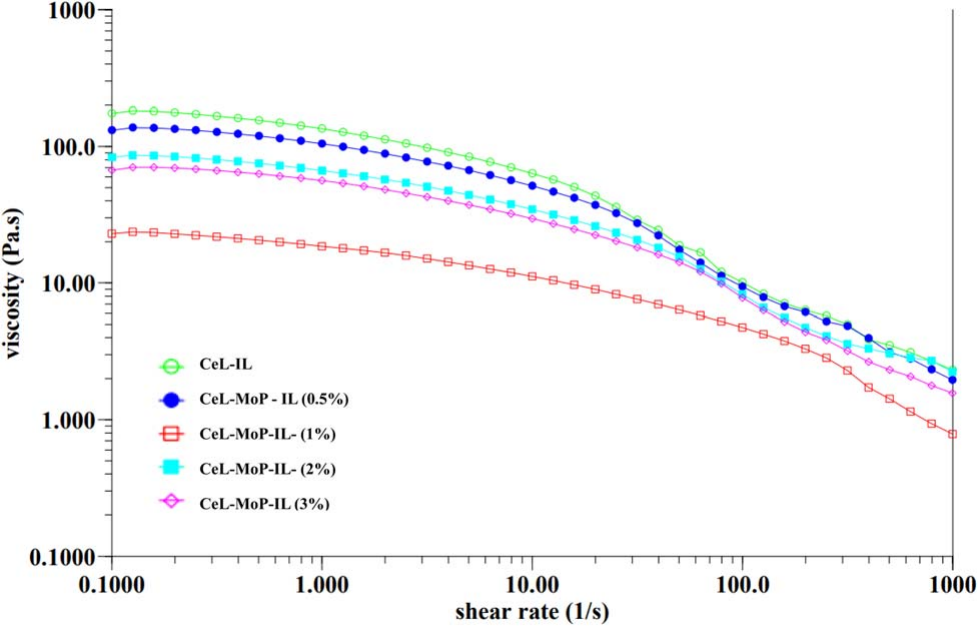
Flow curve with different concentrations of pure CeL–IL and CeL–MoP–IL solutions.

The rheology of a solution, influenced by the choice of solvent, including ionic liquids, plays a crucial role in determining spinnability during the wet spinning process.^47,48^ This analysis of the rheology of the spinning solutions can also help to understand the processes and control of the MoP introduced into the solutions to enhance the good spinnability of the composites during the wet spinning process. To examine how solution preparation affects fibre spinnability, Fig. 2 presents the rheological properties of cellulose solutions combined with varying concentrations of MoP in spinning dopes. These curves highlight the interactions between pure cellulose solutions and their composites, illustrating how these interactions influence the spinnability of fibres. The addition of 0.5% MoP in the ionic liquid resulted in a marginal viscosity reduction of CeL–MoP–IL (0.5%). A further increment of MoP of 1% resulted in a significant reduction in the viscosity of CeL–MoP–IL (1%); this phenomenon might be linked to contents in the MoP, such as protein, carbohydrates, flavonoids, fatty acids, glucosinolates, oils, and some present minerals.^34^ Similar trends were observed in the rheological behaviour of cellulose/silk fibroin blend solutions with ionic liquid as solvent. Furthermore, there were intractable increases and reductions in CeL–MoP (2%) and CeL–MoP–IL (3%). Further additions of MoP at 2% and 3% concentrations led to variable increases and decreases in viscosity, indicating shear-thinning behaviour, as observed in previous literature.^49–52^ This result agrees with previous studies on the rheological properties of solutions of non-woven fabric made from fine regenerated cellulose fibres produced using a wet solution blow spinning method from an ionic-liquid solution and other studies on the structural and property changes in regenerated cellulose fibres caused by the presence of metal ion impurities.^48,52^ Furthermore, our findings confirm the phenomenon of Newtonian fluid plateau properties of minimal shear rates, resulting in a stable solution structure during the flow process, which helps to facilitate the manufacturing process of polymers, including wet spinning fibres at 65 ± 1 °C used for the wet spinning process when the ionic liquid was used for fabrications.^49^ These results indicate that MoP incorporation increases spinning solution instability, negatively impacting the mechanical properties of cellulose fibres. However, the successful integration of MoP into the cellulose fibre matrix suggests potential enhancements that could improve the material's suitability for our proposed applications.

### 3.2 Fourier transform infrared (FTIR) analysis of fibres

**Fig. 3:**
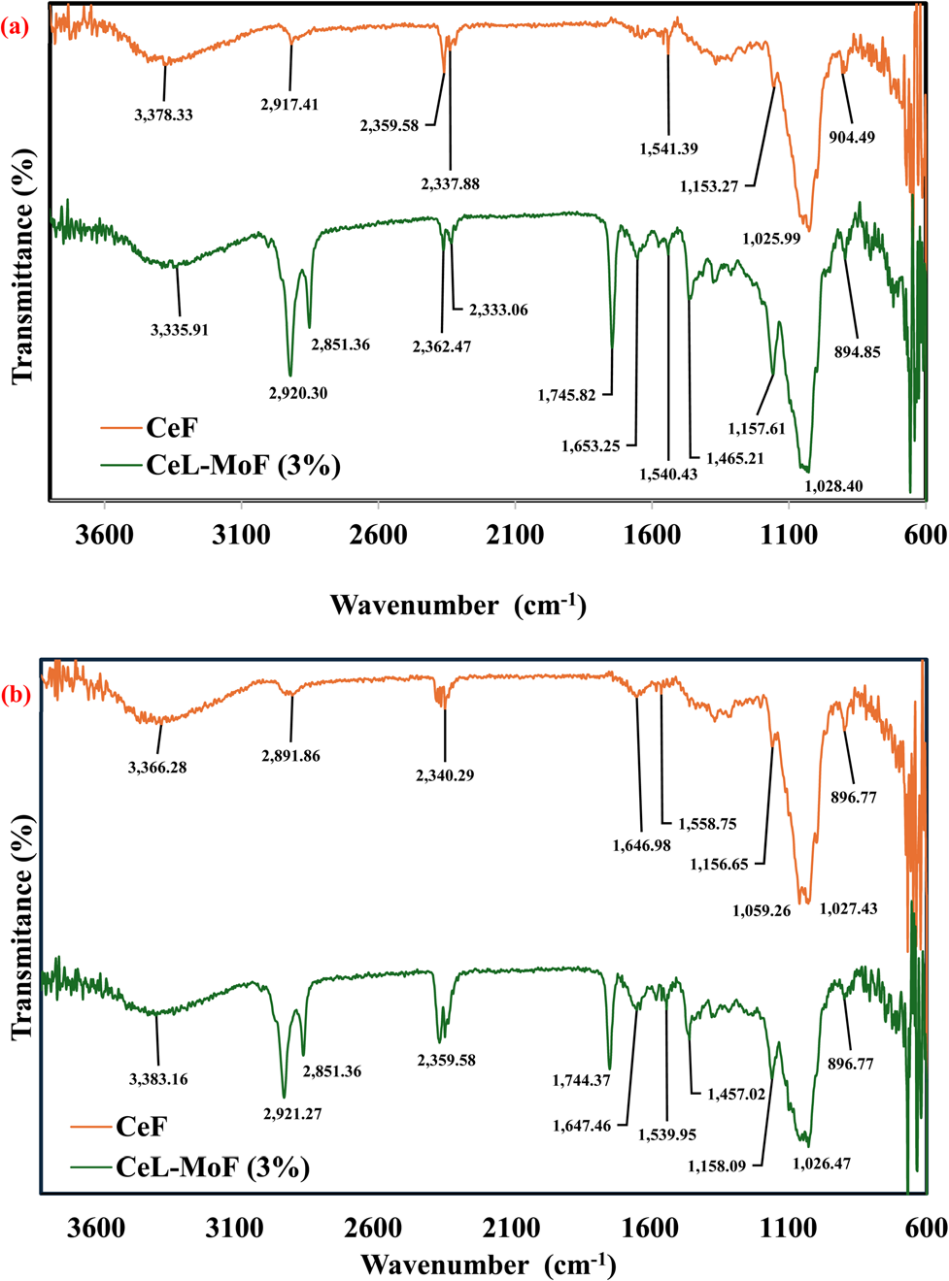
FTIR spectra of CeF (a) before adsorption and (b) after adsorption of heavy metals in aqueous solution and CeL-MoF (3%) composite fibres.

Fig. 3 shows the FTIR spectra of pure cellulose (CeL) and the MoP-loaded composite (CeL-MoF 3%) before and after immersion in aqueous solutions containing Cu^2+^, Cd^2+^, and Ni^2+^ ions. The 3% MoP concentration was selected owing to its superior spectral resolution, functional group diversity, and consistent performance. Spectral comparisons demonstrate key shifts and intensity variations associated with metal ion adsorption. FTIR profiles for additional MoP concentrations (0.5%, 1%, and 2%) are presented in figure in the ESI.†

From the FT-IR spectra shown in Fig. 3a, the pure cellulose exhibited characteristic peaks at 3377.33 cm^−1^ (O–H stretching), 2917.41 cm^−1^ (C–H stretching), and 1025.99 cm^−1^ (C–O–C stretching), which are consistent with literature values for regenerated cellulose.^53–55^ The spectral bands of the pure cellulose fibres predominantly occur within the wave number regions of 3750–2800 cm^−1^ and 1750–600 cm^−1^. The spectra in these regions correspond to FTIR data previously reported for cellulose fibres derived from various wood pulp derivatives.^56^ The peaks observed in the wave number range of 3750–3000 cm^−1^ are attributed to the stretching vibrations of the O–H and C–H bonds typical of polysaccharides. The broad peak at 3378.33 cm^−1^ is characteristic of hydroxyl group stretching in polysaccharides, while the sharp and intense peak at 2917.41 cm^−1^ corresponds to the C–H stretching vibration of hydrocarbon (CH_2_) moieties within the polysaccharide structure.^55^ Furthermore, sharp peaks in the 1550–1750 cm^−1^ range are assigned to the C=O stretching vibrations of the carbonyl linkages present in the fatty acid constituents of Moringa seed extract.^34^ These peaks exhibited increased intensity upon the addition of MoP. The peak at 1465.21 cm^−1^ is attributed to the O–H bending vibration, while the characteristic peak at 1028.40 cm^−1^ correlates with C–O bond stretching. With the incorporation of MoP, several new absorption peaks emerged, and notable shifts in the existing peaks were also observed. For example, a prominent peak at approximately 1653.25 cm^−1^ attributed to the C=O stretching vibrations of proteins and other organic compounds present in MoP^57,58^ became increasingly pronounced with higher MoP concentrations. In addition, a slight shift in the O–H stretching peak was observed, suggesting the formation of hydrogen bonds between the hydroxyl groups of cellulose and the functional moieties in MoP. Additionally, new sharp and intense peaks at 2920.30 cm^−1^ and 2851.36 cm^−1^ correspond to the enhanced asymmetric and symmetric C–H stretching vibration of hydrocarbon (CH_2_ and CH_3_) moieties within the CeL-MoF composite structure. Similar spectral shifts were reported by Zhang *et al.*^59^ In their study, they investigated the incorporation of grape seed extract into regenerated cellulose films. These FTIR spectral modifications collectively suggest that MoP is successfully integrated into the cellulose matrix and that hydrogen bonding interactions occur between the functional groups of MoP and cellulose. This interpretation aligns with the findings of Castro-López *et al.*^60^ who observed comparable peak intensity reductions and shifts following the incorporation of *M. oleifera* seed extract into carboxymethyl cellulose. Specifically, a small signal at 1540.43 cm^−1^ was assigned to the N–O stretching vibration of amino compounds, while a band at 1745.82 cm^−1^ was attributed to the C=O stretching of *M. oleifera*. In the present study, the intensity of the newly formed peaks increased as the MoP content increased, thereby confirming the presence and distribution of MoP within the cellulose fibres.

After the immersion of the fibres into the aqueous heavy metal solution, the adsorption of Cu^2+^, Cd^2+^, and Ni^2+^ ions onto the regenerated composite fibres was assessed through variations in the FTIR spectrum, as shown in Fig. 3b. The characteristic functional groups of cellulose and MoP—namely hydroxyl (−OH), carboxyl (−COOH), amine (−NH_2_), and glycosidic (C–O–C) linkages—are theoretically known to exhibit vibrational frequency shifts upon interaction with metal ions.^61–63^

#### 3.2.1 Hydroxyl (−OH) groups

The O–H stretching vibration, typically occurring as a broad peak in the 3300–3500 cm^−1^ range, shifted to higher wavenumbers (from 3335.91 cm^−1^ to 3383.16 cm^−1^) and showed reduced intensity following metal ion adsorption, as shown in Fig. 3a. This observation indicates hydrogen bonding or coordination interactions between hydroxyl groups and metal ions.

#### 3.2.2 Carboxyl (–COOH) groups

The C=O stretching vibration, normally observed around 1700–1750 cm^−1^, is generally expected to shift toward a lower wavenumber upon the formation of metal-carboxylate complexes. This shift demonstrates metal–oxygen interactions that weaken the C=O bond. However, in this study, the peak around 1744.37 cm^−1^ did not shift, implying that carboxylate coordination was absent or significantly hindered.

#### 3.2.3 Amino/amide (–NH_2_, C=O–NR′R′′) groups

The N–H stretching band observed in the region of 3200–3500 cm^−1^ and the amide I band (C=O–NR′R′′ stretching) near 1647.46 cm^−1^ are also expected to shift following metal ion adsorption. These changes may be attributed to the coordination between the metal ions and nitrogen atoms in the amino or amide groups of MoP. However, the lack of this shift indicates that the CeL-MoF compound does not provide active amino/amide groups for metal coordination compound formation.

#### 3.2.4 Glycosidic (C–O–C) linkages in cellulose

The C–O–C stretching vibrations, situated between 900 and 1100 cm^−1^, showed intensity variation post-adsorption, suggesting interaction between metal ions and ether oxygen atoms in the cellulose backbone. This is particularly evident at the 1027.43 cm^−1^ and 896.77 cm^−1^ peaks.

These spectral shifts collectively provide evidence of metal ion adsorption onto the fibre, where the metal ions form coordination bonds with the oxygen or nitrogen atoms in the functional groups of cellulose and *M. oleifera* seed powder fibre. This is supported by studies conducted by Acheampong *et al.*^64,65^ and Meneghel *et al.*,^66,67^ who found that their FTIR spectra analysis revealed the presence of many functional groups that can bind metal ions by removal using ion exchange. The observed spectral shifts, disappearance, and increased intensity of some peaks after sorption experiments indicate an interaction between metal ions and the *M. oleifera* seed. C=O, amino groups, and –NH_2_ were involved in sorption by MoP. The resulting FTIR data suggest that structural modifications in the functional groups due to CeL–MoP interactions may block or limit some active metal binding in the CeL-MoF composites, thus impeding efficient coordination complex formation with heavy metal ions. This phenomenon may cause the observed preferential adsorption of metals such as Cu^2+^ compared to Cd^2+^ and Ni^2+^, potentially indicating a degree of selectivity in the fibre's binding behaviour.

### 3.3 Thermogravimetric analysis of CeF and CeL-MoF

**Fig. 4:**
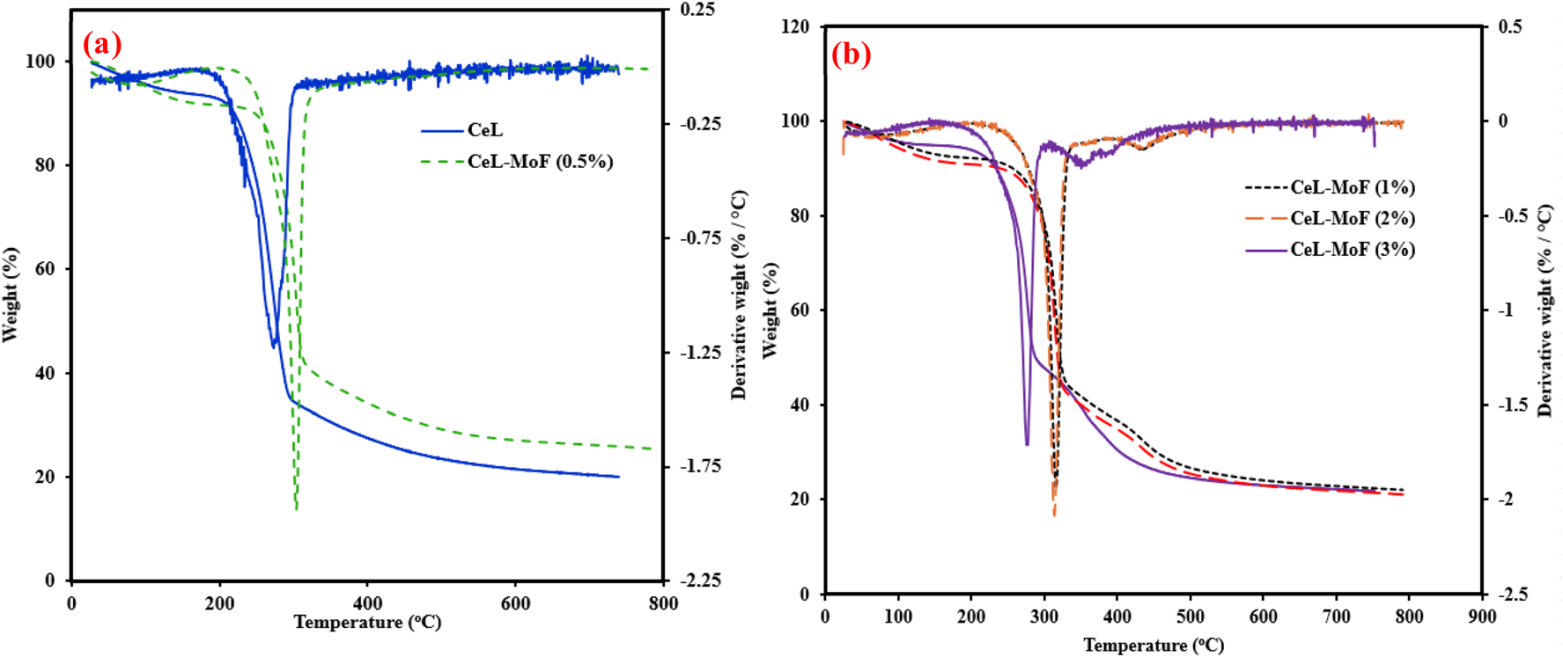
Combined TGA–DTG curve of (a) CeF and CeL-MoF (0.5%); (b) CeL-MoF at different concentrations.

The TGA of CeF and CeL-MoF was conducted, and the combination of the TGA–DTG curve is shown in Fig. 4. As shown in Fig. 4a, the initial temperature of the pure cellulose fibre is approximately 212 °C, while the final decomposition temperature of the CeL reaches a peak of about 342 °C. This is similar to the results obtained in the literature.^41^ The onset degradation temperature of the composites is similar, with little variation in the degradation of each of those composites in the range of 249–243 °C. However, changes were observed in the endset thermal degradation properties of the composites, as shown in Fig. 4. With CeL-MoF (0.5%) degrading at about 314 °C, CeL-MoF (1%) at 325 °C, and CeL-MoF (2%) and (3%) at approximately 465.6 °C and 322.4 °C, respectively. Similar trends were reported in the characteristics of cellulose isolated from the selected biomass, as described by Zhang *et al.*^41^ The thermogravimetric (DTG) curve of the samples also reflects more on the degradation profiles of the fibre samples, with the quantitative summary presented in Table 2. The CeL fibre shows a single sharp peak observed at a temperature of 272.7 °C, which corresponds to the composition stage primarily driven by cellulose breakdown, as reported in the literature.^41,68^ With little addition of CeL-MoF (0.5%), the shift in peaks to approximately 303.06 °C resulted in traces of MoP in the matrix. However, the further addition of MoP in CeL-MoF composites resulted in a shift in peaks of CeL-MoF (1%), which presented two distinct peaks at 316.08 °C and 394.9 °C, respectively, resulting in a sequential thermal decomposition of the cellulose matrix and MoP, respectively. This may be ascribed to the presence of the major constituents of MoP, such as protein, as the content of the MoP increases to 2%, and the second peak shifts to a higher temperature of 427.55 °C, but the profile on the DTG profile appears to overlap with the thermal profile. The CeL-MoF (3%) reflects complex degradation that presents multiple peaks at 276.04 °C, 350.79 °C, and 377.81 °C. These peaks correspond to the thermal decomposition of both the CeL and the combination of the MoP composites, and their synergistic interactions within the composite, as similar trends were observed in studies conducted by Orisawayi *et al.*^10^ when MoP was loaded into a sodium alginate matrix. Conclusively, the overall study of the TGA and the DTG analysis demonstrates that MoP was incorporated into the CeL matrix, which significantly altered the thermal stability and degradation properties of the composites. Conclusively, the deduction from the TGA–DTG studies demonstrated that the degradation of the composites of MoP at higher concentrations occurred in a wide temperature range over time.

**Table 2:** Thermal degradation temperatures of the fibre samples

Sample	*T*_5%_ (°C)	*T*_10%_ (°C)	*T*_50%_ (°C)	*T*_1peak_ (°C)	*T*_2peak_ (°C)	*T*_3peak_ (°C)
CeL	111.93	219.31	275.36	272.70	—	—
CeL-MoF (0.5%)	102.22	247.15	305.38	303.06	—	—
CeL-MoF (1%)	108.47	257.04	321.71	316.08	394.90	—
CeL-MoF (2%)	91.5	240.37	318.21	313.92	427.55	—
CeL-MoF (3%)	136.52	233.65	287.96	276.04	350.79	377.81

### 3.4 SEM structure morphology of CeL and CeL–MoP composite fibres

**Fig. 5:**
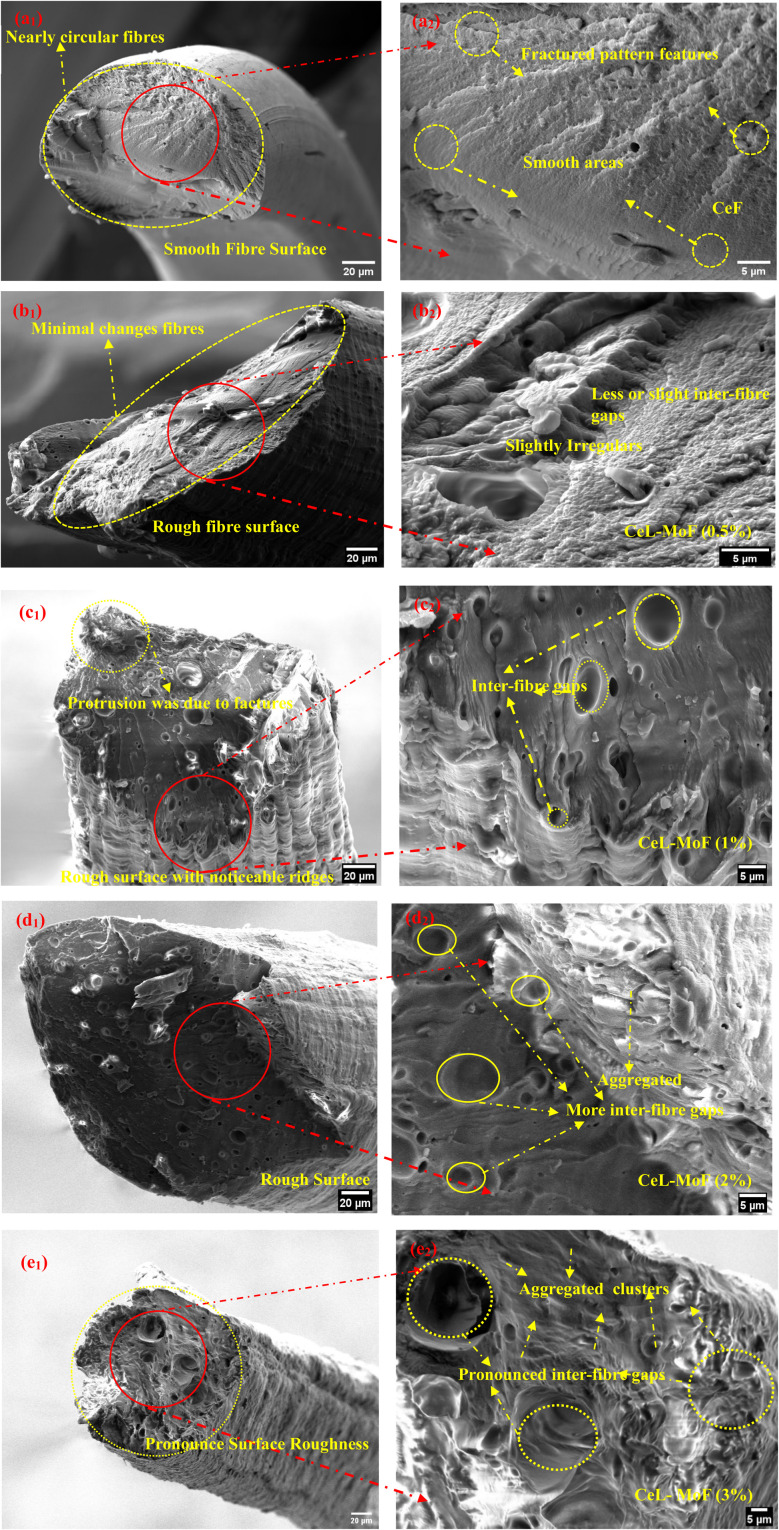
Structural morphology and magnified cross-sections of wet-spun fibres: CeL ((a_1_) surface morphology, (a_2_) cross-section), CeL-MoF (0.5%) (b_1_ and b_2_), CeL-MoF (1%) (c_1_ and c_2_), CeL-MoF (2%) (d_1_ and d_2_), and CeL-MoF (3%) (e_1_ and e_2_). The images show surface details and internal structural changes as the MoP content increases.

The structural morphology and cross-sectional views of the wet-spun fibre samples are presented in Fig. 5. The images of the pure cellulose fibre (CeF) are shown in Fig. 5a_1_ and a_2_. CeF reveals a smooth, uniform surface morphology, consistent with the characteristics of no visible inter-fibre gaps on the surface of pure regenerated cellulose fabricated from IL, as reported in previous studies.^51,69^ The cross-sectional view of the CeF exhibits a near-circular structure with a smooth and well-defined surface. Internally, the fibres appear homogenous, indicating the high quality of the wet-spinning process.^10,69,70^ The magnified cross-section in Fig. 5a_2_ features fracture patterns that do not seem to originate from inherent defects. This demonstrates the excellent structural integrity of the CeF. These characteristics establish CeF as a reliable baseline for further material modifications or the incorporation of additives, such as MoP.

Fig. 5b_1_–e_2_ shows the surface morphology of CeL-MoF fibres at various MoP concentrations. The CeL-MoF (0.5%) composite in Fig. 5b_2_ presents a well-dispersed MoP particle distribution within the cellulose matrix. The surface appears slightly irregular compared to the CeF fibres. There were features of few or minimal processing and intrinsic defects observed on the surface of the fibres. These features are consistent with prior studies on low MoP loading in biopolymer matrices, such as alginate, as noted by Orisawayi *et al.*^10^

CeL-MoF (1%) composites (Fig. 5c_2_) exhibit a rougher surface than CeL-MoFs (0.5%), with features resembling the ridges on the surface of the fibres. These features differ from those observed in CeL fibres and the CeL-MoF (0.5%) composite. Protrusions were also observed on the surface during fractures, suggesting modifications caused by MoP incorporation that must have resulted from the factored surfaces. As the concentration of MoP increases to 2%, as illustrated in Fig. 5d_2_, the CeL-MoF (2%) composite fibres exhibit more pronounced surface irregularities, with visible MoP particles and increased inter-fibre gaps with aggregations. The morphology shows moderate roughness, disrupting the previously smooth structure. The pronounced ridges on the surface seam disappear but are slightly observed.

In the CeL-MoF (3%) composite shown in Fig. 5e_2_, features of significantly pronounced inter-fibre gaps were observed compared to other composites of CeL-MoF and CeF fibres. The surface displays a high concentration of defects, with noticeable aggregations of MoP at these concentrations disrupting uniformity, which must have affected the mechanical properties. The cross-sectional view reveals severe inter-fibre gaps and considerable heterogeneity, suggesting that excess MoP loading alters the cellulose matrix. This could be attributed to interactions between the bioactive compounds in MoP and the cellulose matrix, which affect the structural integrity of the fibres during the coagulation process.

Previous studies have reported increased surface roughness in fibres due to MoP incorporation into biopolymers. This phenomenon is often linked to interactions between bioactive compounds in MoP and the polymer matrix, which disrupt homogeneity and contribute to surface irregularities, as noted by Orisawayi *et al.*,^10^ Coscia *et al.*,^71^ and Yang *et al.*^72^

The findings of this study are consistent with these observations, showing a significant increase in the MoP content of approximately 8% when incorporated into alginate biopolymers.^10^ This increase prevents further loading, as it leads to greater morphological disruptions and inter-fibre gaps. Similarly, our findings indicate that during preparation, the agglomeration of MoP is minimized, preventing the further addition of MoP into the CeL matrix.

In conclusion, higher MoP concentrations significantly affect fibre morphology, increasing surface roughness, inter-fibre gaps, and heterogeneity. The 3% MoP sample exhibits the most pronounced changes, with aggregated MoP clusters causing structural disorders. These results highlight the impact of MoP loading on the cellulose matrix, providing valuable insights for optimising composite material properties.

### 3.5 Atomic force microscopy

**Fig. 6:**
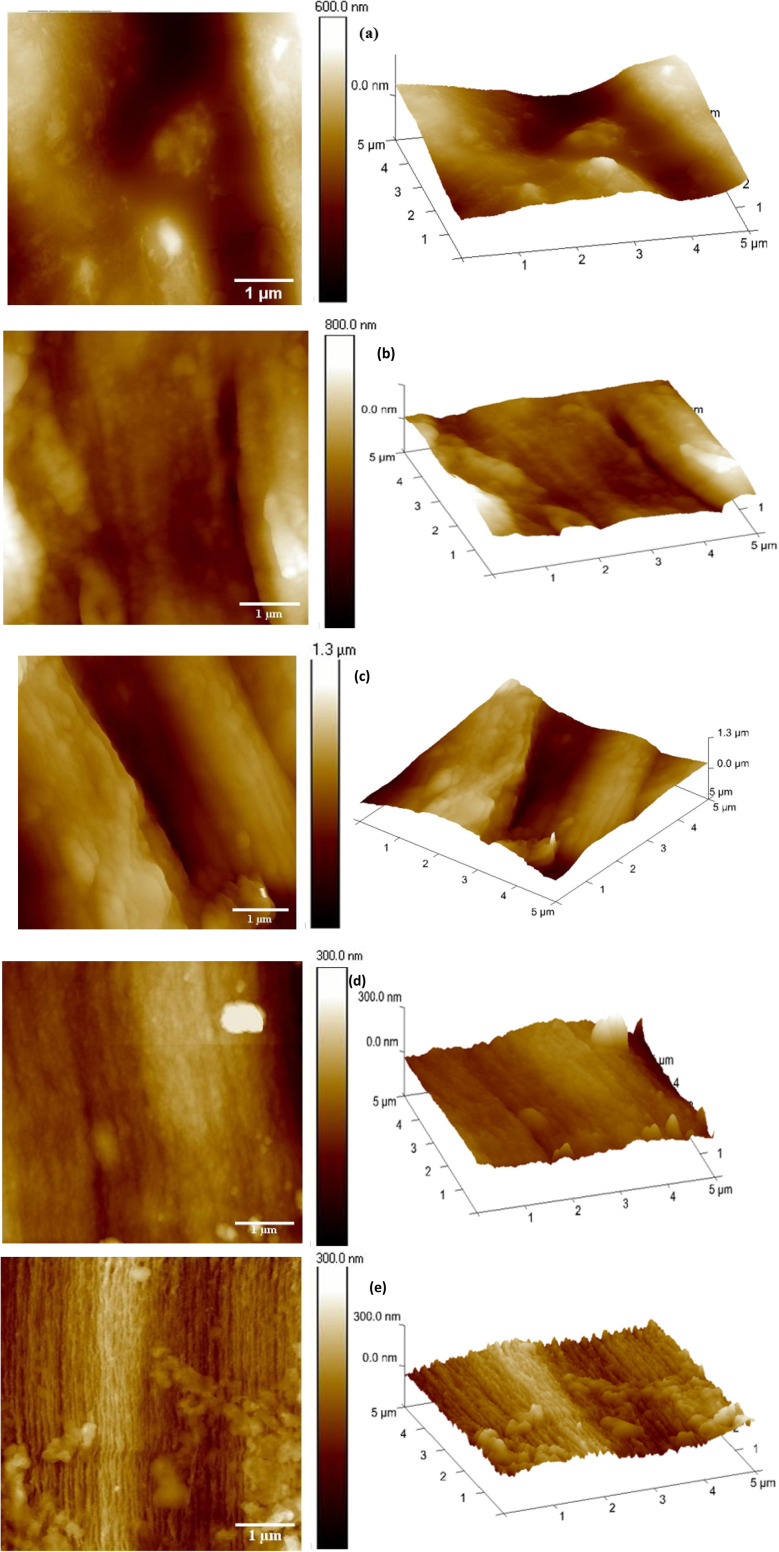
AFM topography of 2D and 3D surface maps are presented for (a) CeF, (b) CeL-MoF (0.5%), (c) CeL-MoF (1%), (d) CeL-MoF (2%), and (e) CeL-MoF (3%), showing the surface structural variations of the composites.

Over a few decades, Atomic Force Microscopy (AFM) has been employed as a multipurpose analytical technique and has proven to be extremely useful in characterising complicated topographic images of materials from micrometres to nanometres.^73,74^ To the best of our knowledge, only few studies have reported the use of AFM to characterise the nature of the microscopic topographic image of wet-spun fibre. The 2-D and 3-D topographic images of the CeL and CeL-MoF composite samples obtained are illustrated in Fig. 6. The roughness parameters, arithmetic mean roughness (*R*_a_), root mean square roughness (*R*_q_), and maximum roughness depth (*R*_max_) are commonly accepted and frequently used to determine roughness measurement.^75^Table 3 presents the quantitative results from the AFM surface roughness properties of the CeF and CeL-MoF fibres. The (*R*_a_) and (*R*_q_) values for pure CeL fibre were 23.7 nm and 29.2 nm, respectively. This is an attribute of CeF, presenting a smooth surface topology, as observed in Fig. 6a. This illustration shows a moderate level of surface roughness structures compared to CeL-MoF composite fibres. These features could be evidence of the complete dissolution of the CeF fragments in IL, which is crucial for excellent fibre formation during the wet spinning process. This finding aligns with studies on the common characteristics of pure cellulose, where a similar topology was observed in fibres produced through dry-jet wet-spinning.^76^ As depicted in Fig. 6b–e, the incorporation of MoP at different concentrations of 0.5%, 1%, 2% and 3% wt. resulted in increments in *R*_a_ and *R*_q_ values to 30.6 and 42.2, 75.1 and 92.5, 79.1 and 104, as well as 175 and 207 nm, respectively. This rapid increase in the value of *R*_max_ from 603 nm to about 1181 nm, nearly doubling the value, resulted in a noticeable change, as shown in Fig. 6e. Thus, the surface's topography can be modulated to increase the surface area, as observed in Fig. 6e. Although this change influences the mechanical properties, it strongly indicates the successful distinctive feature attributed to the incorporation of MoP into the CeF matrix composites.

**Table 3:** AFM Surface roughness properties of the CeF and CeL-MoF fibres

Fibre Code	*R*_a_ (nm)	*R*_q_ (nm)	*R*_max_ (nm)
CeF	23.7	29.2	200
CeL-MoF (0.5%)	30.6	42.2	386
CeL-MoF (1%)	75.1	92.5	464
CeL-MoF (2%)	79.1	104	603
CeL-MoF (3%)	175	206	1181

### 3.6 Fibre diameter and mechanical properties of CeF and CeL-MoF

**Fig. 7:**
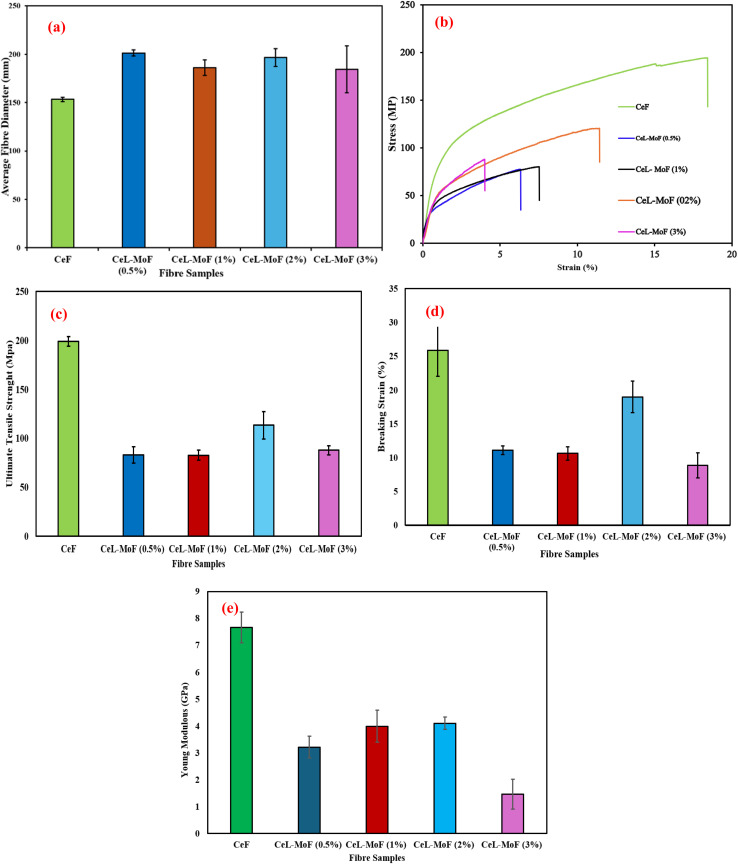
(a) Average fibre diameter, and the mechanical properties of CeF and CeL-MoF composite fibres: (b) stress–strain curve, (c) breaking strain, (d) ultimate tensile strength, and (e) Young's modulus for carbohydrate polymer applications.

The fibre diameter and the mechanical properties are important in determining the behaviour of wet-spun fibre.^75^Fig. 7 shows the CeL and CeL-MoF composite's average diameter properties and the mechanical properties of CeF and CeL-MoF composite fibres with the stress–strain curve, ultimate tensile strength, breaking strain, and Young's modulus.

The fibre diameter variations during wet spinning, influenced by factors such as filler dispersion, particle concentration, spinning speed, and drying conditions, significantly affect the mechanical properties, explaining why they change and how this facilitates the incorporation of MoF.^70^Fig. 7a illustrates these variations and their impact on CeF and CeL-MoF fibres. Regarding mechanical properties, the control sample, CeF, exhibited the highest values (Fig. 7b–e), with UTS of approximately 199.1 MPa (±4.76), breaking strain of 25.9% (±3.88), and Youngs' Modulus (YM) of 7.66 GPa (±0.57), demonstrating the intrinsic strength and flexibility of pure regenerated CeL fibres developed in this study. In comparison, the mechanical properties of CeL-MoF composites varied with MoP concentration, particularly MoP at 0.5% and 1%, possessing similar trends.

In detail, the introduction of 0.5 MoP reduces the UTS of the CeL-MoF to approximately 83.23 MPa (±8.31), which is significantly lower than that of the CeF. This reflects the initial impact of MoP incorporated on the CeF fibre's mechanical properties, with a breaking strain of 11.07% (±0.65). This impact was observed to reduce the flexibility of pure CeF. The YM also substantially decreased compared to 3.21 GPa (±0.4) compared to that of CeF, which may be due to disruption in its molecular arrangement, leading to a decline in mechanical properties, even at low concentrations of MoP. These results may be related to the results of the study conducted by Ejeta *et al.*^77^ on the influence of filler concentrations and processing parameters on the mechanical properties of lignocellulose loaded with bio-fillers. The CeL-MoF (1%) UTS is similar to that of MoP at 0.5% with little improvement to about 82.74 MPa (±5.16) at this concentration, while the breaking strain is 10.63% (±1), showing a further reduction in flexibility at this trend; the YM is approximately 3.99 GPa (±0.6), demonstrating a slight improvement compared to 0.5% MoP. This change is potentially due to improved particle dispersion during solution preparation with IL or interactions between bioactive compounds in MoP, such as proteins, lipids, fatty acids, or antioxidants, which may weaken the cellulose structure.^77,78^

Interestingly, the fibres of the CeL-MoF (2%) composites show a UTS of approximately 113.42 MPa (±14.09), which is an improvement over the 0.5% and 1% MoP composites, suggesting optimal alignment of MoP particles at this concentration. The result obtained from the breaking strain is about 18.98% (±2.32), which is significantly higher than other composites, indicating improved ductility. The YM of 4.1 GPa (±0.23) is the highest among the composites, but there is approximately a 43.0% reduction in UTS and a 46.5% reduction compared to the CeF control. This improvement can be compared with studies by Coscia *et al.*^79^ on the particle dispersion of curcumin actively loaded on regenerated cellulose, thereby improving the mechanical properties of the composites at a certain threshold. Furthermore, CeL-MoF (3%) with a UTS of approximately 87.97 MPa (±4.66), a decrease compared to 2% MoP, can be attributed to particle agglomeration, causing structural defects. This decrease in tensile strength aligns with the SEM and AFM analyses, which reveal increased surface roughness at higher MoP concentrations. The breaking strain at this concentration was 8.86% (±1.84), which is the lowest among the composites, indicating increased brittleness, with a YM of 1.46 GPa (±0.55), which was drastically reduced, highlighting the adverse effects of agglomeration at higher MoP concentrations.

Conclusively, the key finding from the result of the mechanical test of our composites was that the mechanical properties of the composites were lower than those of our CeF owing to varied concentrations of MoP. Our study finally shows that among the composites, CeL-MoF at 2% MoP demonstrated the best mechanical performance with a breaking strain of 18.98%, which was significantly reduced to 8.86%, which is a higher concentration of MoP of 3%. This progressive decrease could be attributed to the earlier stated weakness in the interface bonding caused by the agglomeration of the polysaccharide matrix associated with the loading of MoP particles reported by Orisawayi *et al.*^10^ Studies on alginate polysaccharide matrix loaded with MoP at 8% could also be related to agglomeration disrupting the cellulose structure, causing more brittleness at this concentration, possibly associated with easier crack propagation within the structures of previously reported polysaccharide composites.^80,81^

### 3.7 SEM-energy dispersive X-ray spectroscopy (EDX) of CeF and CeL-MoF samples

**Fig. 8:**
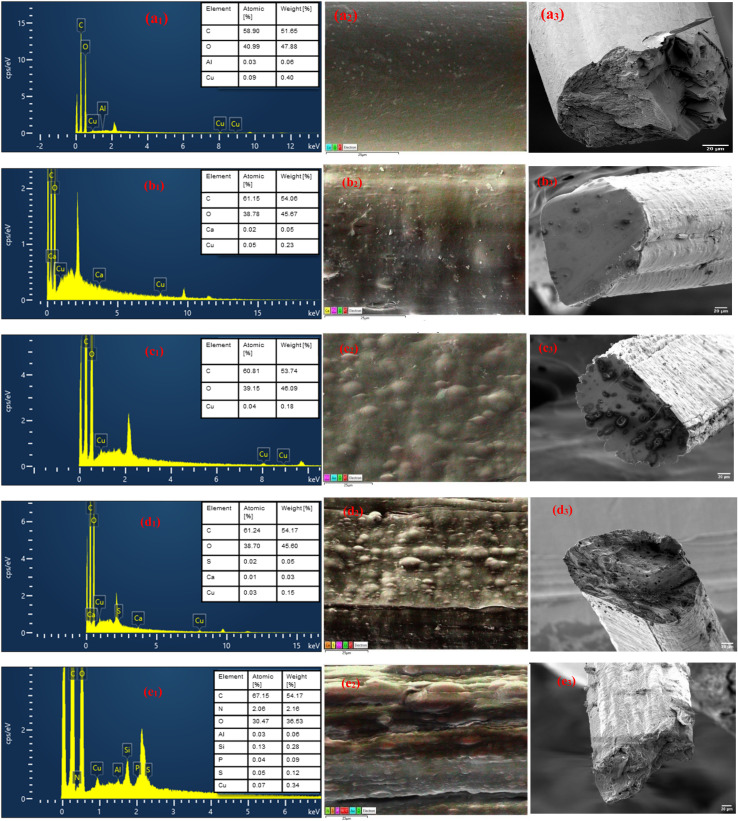
SEM-EDX analysis of composites: elemental peaks (a_1_–e_1_), fibre surface morphology (a_2_–e_2_), and cross-sectional morphology (a_3_–e_3_) for (a) CeFs, (b) CeL-MoFs (0.5%), (c) CeL-MoFs (1%), (d) CeL-MoFs (2%), and (e) CeL-MoFs (3%) after (Cu^2+^, Ni^2+^, and Cd^2+^) adsorption from the solution.

The analyses of the CeF and CeL-MoF samples were performed. The key elements used are the same and agree with our previous studies.^10^ The spectra from this analysis are presented in Fig. 8, showing the presence of common elements, such as carbon and oxygen, associated with CeL and MoP, respectively.^82,83^

Additional elements such as aluminum, calcium, sulphur, and silicon are characteristic of MoP, as reported in previous studies.^10^ The immersed fibre spectrum of the CeL-MoF presented an additional peak related to that of MoP content, consistent with the increasing concentration of MoP in the composite fibres. This was observed in the pure samples. This is corroborated by the observations made in previous studies on the use of EDX analysis on *M. oleifera* seed derivatives, as reported by Acheampong *et al.*,^64^ Benettayeb *et al.*,^84^ Mateus *et al.*,^85^ Belbali *et al.*,^86^ and Okoya *et al.*^87^ Compared with our FTIR findings, this provides visual and elemental evidence of the successful incorporation of MoP into the cellulose matrix.

**Fig. 9:**
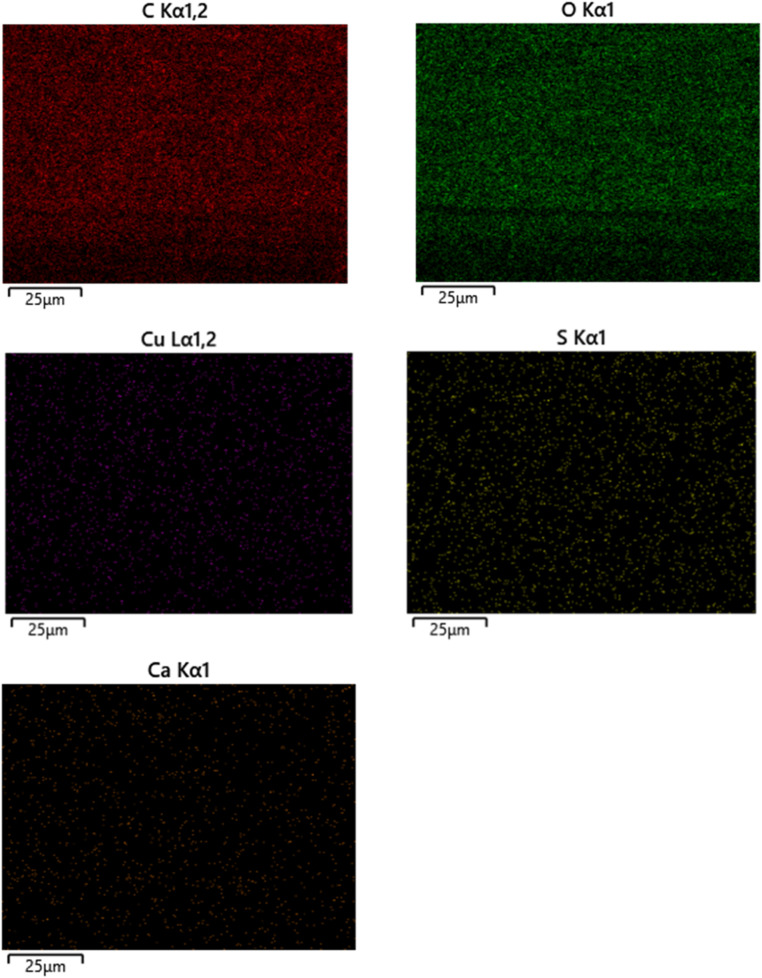
SEM-EDX mapping of CeL-MoF (2%) composites.

The adsorption of heavy metals was also validated using EDX as a qualitative measure, where copper (Cu^2+^) has shown the most affinity for adsorption through the CeL-MoF composite fibres. Nickel (Ni^2+^) and cadmium (Cd^2+^) are not visible on such spectra, indicating that they were present in trace amounts and could not be detected by the sensors. Similar studies conducted by Acheampong *et al.*,^64^ on adsorption of Cu(ii) using *M. oleifera* seed powder, inferred that there is an involvement of ion exchange mechanisms in the biosorption process for the removal of copper by *M. oleifera* seeds by the –C=O group. However, Ni^2+^ and Cd^2+^ ion absorption mechanisms could have been hampered by a competitive sorption environment due to the multi-metal presence in the test solution. In addition, the literature suggests^88–90^ that the sorption affinity of the metal ions depends on the atomic weight, electronegativity, electrode potential, and ionic size. Matouq *et al.*^90^ demonstrated, for raw *M. oleifera* seed powder, that Cu^2+^ has a high affinity for sorption in a solution with Ni^2+^, Cr^3+^, Cu^2+^, and Zn^2+^. This aligns with our results, explaining why other heavy metals, such as Ni^2+^ and Cd^2+^, were not visible in the spectra. Ni^2+^ and Cd^2+^ were not visible in the spectra. This preliminary analysis highlights the capacity of the developed composites to capture heavy metals. The EDX mapping illustrated in Fig. 9 presents the CeL-MoF (2%). The mapping shows that the metal ions after immersion are uniformly distributed and homogeneous within the CeL-MoF matrix, which is consistent with previous studies,^10^ indicating similar effective and uniform adsorption related to studies conducted by Orisawayi *et al.*, 2024. The deduction from these studies shows that composites can capture heavier metals compared to CeF. Thus, CeL-MoF composites have significant potential for removing heavy metals from water.

## 4. Conclusion

This study demonstrates the novel fabrication of CeL-MoF composite potential materials for the selective adsorption of heavy metal ions from an aqueous solution. The *M. oleifera* seed, identified as a natural biosorbent, affects the mechanical properties of cellulose in the developed composites owing to the incorporation of MoP and affects adsorption performance. FTIR and SEM-EDX analyses confirm the successful integration of MoP into the cellulose matrix. CeL-MoF (2%) exhibits improved mechanical properties and enhanced adsorption efficiency compared to pure CeF. The results from the rheology confirm that the solution is suitable for spinning, and the TGA–DTG results confirm variations in the properties of the developed CeL-MoF through the demonstration of variations in the thermal properties. Notably, the preliminary study confirmed that the composites have more affinity towards the adsorption of copper (Cu^2+^). The MoP concentration exhibited an optimal balance between the mechanical properties and adsorption performance, highlighting the unique alignment of MoP particles within the matrix. The use of ionic liquids in wet spinning provides a scalable, environmentally friendly fabrication method for dissolving cellulose, which can offer an alternative method to help in fabrication compared to conventional membrane technologies. This work addresses critical gaps in heavy metal adsorption by presenting a sustainable, cost-effective solution that aligns with green chemistry principles. Future work will focus on optimisation of the CeL-MoF composites by exploring more characterisation and evaluating properties such pore size distribution using the BJH (Barrett–Joyner–Halenda) method, DFT (Density Functional Theory), viscoelasticity, fibre-matrix interactions and stimulating the composite to determine properties such as adsorption capacity, sorption kinetics, and thermodynamics using models such as Langmuir, Freundlich, Temkin, and pseudo-second-order, to explore its selectivity, considering additional metals such as Fe, As, and other mutagenic agents from the wastewater. Furthermore, we will consider the scalability of our Cel-MoF fibres for practical water purification applications, ensuring that our research contributes to both scientific knowledge and real-world solutions.

## Data availability

The data and supporting documents are available from the corresponding author upon reasonable request.

## Author contributions

Abimbola Oluwatayo Orisawayi: formal analysis, conceptualization, investigation, methodology, funding acquisition, project administration, investigation, methodology, software, visualization, writing the original draft, and writing – review & editing. Prithivi Boylla: software, visualization, writing the original draft, and writing – review & editing. Krzysztof Koziol and Sameer S. Rahatekar: supervision, visualization, writing – review & editing, supervision, and resources.

## Conflicts of interest

The authors declare that they have no known competing financial interests or personal relationships that could have appeared to influence the work reported in this paper.

## Supplementary Material

RA-015-D5RA02386F-s001

## References

[cit1] Sayyed A. J., Pinjari D. V., Sonawane S. H., Bhanvase B. A., Sheikh J., Sillanpää M. (2021). J. Environ. Chem. Eng..

[cit2] Musa M., Gao Y., Rahman P., Albattat A., Ali M. A. S., Saha S. K. (2024). Clean Technol. Environ. Policy.

[cit3] Venkatraman G., Giribabu N., Mohan P. S., Muttiah B., Govindarajan V. K., Alagiri M., Abdul Rahman P. S., Karsani S. A. (2024). Chemosphere.

[cit4] Dinakarkumar Y., Ramakrishnan G., Gujjula K. R., Vasu V., Balamurugan P., Murali G. (2024). Environ. Chem. Ecotoxicol..

[cit5] Zhang P., Yang M., Lan J., Huang Y., Zhang J., Huang S., Yang Y., Ru J. (2023). Toxics.

[cit6] Dippong T., Resz M.-A., Tănăselia C., Cadar O. (2024). J. Hazard. Mater..

[cit7] Rathi B. S., Kumar P. S., Vo D.-V. N. (2021). Sci. Total Environ..

[cit8] Afroze S., Sen T. K. (2018). Water, Air, Soil Pollut..

[cit9] Zhu F., Zheng Y.-M., Zhang B.-G., Dai Y.-R. (2021). J. Hazard. Mater..

[cit10] Orisawayi A. O., Koziol K., Rahatekar S. S. (2024). Carbohydr. Polym. Technol. Appl..

[cit11] Celebioglu A., Topuz F., Irem Yildiz Z., Uyar T. (2019). ACS Omega.

[cit12] Hendricks D. W. , Water Treatment Unit Processes, CRC Press, Boca Raton, 2018

[cit13] Ince M. and Kaplan Ince O., in Biochemical Toxicology – Heavy Metals and Nanomaterials, ed. M. Ince, O. K. Ince and G. Ondrasek, IntechOpen, 2020, pp. 1–19

[cit14] Li L., Qian X., Shen J. (2022). Carbohydr. Polym..

[cit15] Fakhre N. A., Ibrahim B. M. (2018). J. Hazard. Mater..

[cit16] Pei X., Gan L., Tong Z., Gao H., Meng S., Zhang W., Wang P., Chen Y. (2021). J. Hazard. Mater..

[cit17] Ji F., Li C., Tang B., Xu J., Lu G., Liu P. (2012). Chem. Eng. J..

[cit18] Doyo A. N., Kumar R., Barakat M. A. (2023). J. Taiwan Inst. Chem. Eng..

[cit19] Sánchez J., Butter B., Rivas B. L. (2020). J. Chil. Chem. Soc..

[cit20] Zia Z., Hartland A., Mucalo M. R. (2020). Int. J. Environ. Sci. Technol..

[cit21] Mututuvari T. M., Tran C. D. (2014). J. Hazard. Mater..

[cit22] Hosseini S. F., Nahvi Z., Zandi M. (2019). Food Hydrocolloids.

[cit23] Rahaman M. H., Islam M. A., Islam M. M., Rahman M. A., Alam S. M. N. (2021). Curr. Res. Green Sustainable Chem..

[cit24] Wu Y.-L., Xu S., Wang T., Wang C.-F. (2018). ACS Appl. Mater. Interfaces.

[cit25] Kobayashi S., Sakamoto J., Kimura S. (2001). Prog. Polym. Sci..

[cit26] Oladele I. O., Origbemisoye T. B., Taiwo A. S., Oyegunna S. A., Adelani S. O., Olanrewaju O. F., Orisawayi A. O. (2024). Adv. Mat. Sustain. Manuf..

[cit27] Nagaraja S., Anand P. B., Mohan Kumar K., Ammarullah M. I. (2024). RSC Adv..

[cit28] Kebede T. G., Dube S., Nindi M. M. (2018). Mater. Res. Express.

[cit29] Aderinola T. A., Alashi A. M., Nwachukwu I. D., Fagbemi T. N., Enujiugha V. N., Aluko R. E. (2020). Food Hydrocolloids.

[cit30] Adetuyi F. O., Akintimehin E. S., Karigidi K. O., Orisawayi A. O. (2025). Int. J. Food Sci..

[cit31] Mishra P. P., Mohanty C., Das N., Mishra M., Mohanty A. K., Manna S., Behera A. K. (2024). Water, Air, Soil Pollut..

[cit32] Obuseng V. C., Moshoeshoe M. N., Nareetsile F. M., Kwaambwa H., Maina I. (2022). Front. Chem..

[cit33] Vázquez-Guerrero A., Cortés-Martínez R., Alfaro-Cuevas-Villanueva R., Rivera-Muñoz E., Huirache-Acuña R. (2021). Water.

[cit34] Orisawayi A. O., Koziol K., Hao S., Tiwari S., Rahatekar S. S. (2024). RSC Adv..

[cit35] Ravikumar U., Mater J. (2020). J. Mater. Environ. Sci..

[cit36] Kuznik I., Kruppke I., Cherif C. (2022). Polymers.

[cit37] Dubey S., Bharmoria P., Gehlot P. S., Agrawal V., Kumar A., Mishra S. (2018). ACS Sustain. Chem. Eng..

[cit38] Zhao D., Li H., Zhang J., Fu L., Liu M., Fu J., Ren P. (2012). Carbohydr. Polym..

[cit39] Zhao Z., Wang J., Yuan H., Xu J., Gao H., Nie Y. (2024). ACS Appl. Mater. Interfaces.

[cit40] Qiao T., Yang C., Zhao L., Feng Y., Feng X., Mao Z., Wang B. (2024). Int. J. Biol. Macromol..

[cit41] Zhang Z., Zhu M., Zhang D. (2018). Appl. Energy.

[cit42] Hadou A., Belaadi A., Alshaikh I. M. H., Ghernaout D. (2024). Case Stud. Therm. Eng..

[cit43] Lu H., Butler J. A., Britten N. S., Venkatraman P. D., Rahatekar S. S. (2021). Nanomaterials.

[cit44] Sivalingam A. M., Pandian A. (2024). Carbohydr. Polym. Technol. Appl..

[cit45] Sabapathi N., Ramalingam S., Aruljothi K. N., Lee J., Barathi S. (2023). Plants.

[cit46] Jaekel E. E., Torres G. R., Antonietti M., Rojas O. J., Filonenko S. (2024). Sci. Rep..

[cit47] Nygren N., Schlapp-Hackl I., Heimala S., Sederholm H., Rissanen M., Hummel M. (2024). Carbohydr. Polym..

[cit48] Wei J., Long Y., Wang B., Wu H., Gao H., Nie Y. (2024). Int. J. Biol. Macromol..

[cit49] Lundahl M. J., Berta M., Ago M., Stading M., Rojas O. J. (2018). Eur. Polym. J..

[cit50] Yoo M. K., Reza M. S., Kim I. M., Kim K. J. (2015). Fibers Polym..

[cit51] Ma Y., Nasri-Nasrabadi B., You X., Wang X., Rainey T. J., Byrne N. (2021). J. Nat. Fibers.

[cit52] Zhang J., Kitayama H., Gotoh Y., Potthast A., Rosenau T. (2019). Carbohydr. Polym..

[cit53] Heinze T. , in Advances in Polymer Science, 2015, pp. 1–52

[cit54] Shang W., Sheng Z., Shen Y., Ai B., Zheng L., Yang J., Xu Z. (2016). Carbohydr. Polym..

[cit55] Liu Z., Wang H., Li Z., Lu X., Zhang X., Zhang S., Zhou K. (2011). Mater. Chem. Phys..

[cit56] Hospodarova V., Singovszka E., Stevulova N. (2018). 09. Am. J. Anal. Chem..

[cit57] Araújo C. S. T., Alves V. N., Rezende H. C., Almeida I. L. S., de Assunção R. M. N., Tarley C. R. T., Segatelli M. G., Coelho N. M. M. (2010). Water Sci. Technol..

[cit58] Alghamdi A., Rajan K. P., Thomas S. P. (2024). Case Stud. Chem. Environ. Eng..

[cit59] Zhang Y., Li J., Huang X., Yang C., Wu C., Yang Z., Li D. (2023). Int. J. Biol. Macromol..

[cit60] Castro-López C., Espinoza-González C., Ramos-González R., Boone-Villa V. D., Aguilar-González M. A., Martínez-Ávila G. C. G., Aguilar C. N., Ventura-Sobrevilla J. M. (2021). Food Res. Int..

[cit61] Maina I. W., Obuseng V., Nareetsile F. (2016). J. Chem..

[cit62] Reddy D. H. K., Seshaiah K., Reddy A. V. R., Rao M. M., Wang M. C. (2010). J. Hazard. Mater..

[cit63] Hegazy I., Ali M. E. A., Zaghlool E. H., Elsheikh R. (2021). Appl. Water Sci..

[cit64] Acheampong M. A., Pereira J. P. C., Meulepas R. J. W., Lens P. N. L. (2011). J. Chem. Technol. Biotechnol..

[cit65] Acheampong M. A., Ansa E. D. O., Woode M. Y., Awuah E. (2015). Chem. Eng. Commun..

[cit66] Meneghel A. P., Gonçalves Jr A. C., Strey L., Rubio F., Schwantes D., Casarin J. (2013). Quim. Nova.

[cit67] Meneghel A. P., Gonçalves A. C., Rubio F., Dragunski D. C., Lindino C. A., Strey L. (2013). Water, Air, Soil Pollut..

[cit68] Muddasar M., Menéndez N., Quero Á., Nasiri M. A., Cantarero A., García-Cañadas J., Gómez C. M., Collins M. N., Culebras M. (2024). Adv. Compos. Hybrid Mater..

[cit69] Lee Y. J., Lee S. J., Jeong S. W., Kim H., Oh T. H., Lee S. G. (2019). Fibers Polym..

[cit70] Jeong H. D., Kim S. G., Choi G. M., Park M., Ku B.-C., Lee H. S. (2021). Chem. Eng. J..

[cit71] Coscia M. G., Bhardwaj J., Singh N., Santonicola M. G., Richardson R., Thakur V. K., Rahatekar S. (2018). Ind. Crops Prod..

[cit72] Yang S.-C., Liao Y., Karthikeyan K. G., Pan X. J. (2021). Environ. Pollut..

[cit73] Khan S. A., Ahmed M. A., Baig M. M., Rehman M. M., Yang Y., Lee S. G., Choi J. W., Kim W. Y. (2024). Chem. Eng. J..

[cit74] Zhang H., Huang J., Wang Y., Liu R., Huai X., Jiang J., Anfuso C. (2018). Opt. Commun..

[cit75] Zhang Z., Kong Y., Gao J., Han X., Lian Z., Liu J., Wang W.-J., Yang X. (2024). Nanoscale.

[cit76] Nypelö T., Asaadi S., Kneidinger G., Sixta H., Konnerth J. (2018). Cellulose.

[cit77] Ejeta L. O., Zheng Y., Zhou Y. (2024). Mech. Compos. Mater..

[cit78] Falowo A. B., Mukumbo F. E., Idamokoro E. M., Lorenzo J. M., Afolayan A. J., Muchenje V. (2018). Food Res. Int..

[cit79] Coscia M. G., Bhardwaj J., Singh N., Santonicola M. G., Richardson R., Thakur V. K., Rahatekar S. (2018). Ind. Crops Prod..

[cit80] Sampath P., Santhanam S. K. V. (2019). Polímeros.

[cit81] Vázquez-Guerrero A., Cortés-Martínez R., Alfaro-Cuevas-Villanueva R., Rivera-Muñoz E., Huirache-Acuña R. (2021). Water.

[cit82] Guan F., Tao J., Yao Q., Li Z., Zhang Y., Feng S., Sun J., Yang Q., Song X., Guo J., Liu Y. (2024). Colloids Surf. A Physicochem. Eng. Asp..

[cit83] Ajmal Z., Ali H., Ullah S., Kumar A., Abboud M., Gul H., Al-hadeethi Y., Alshammari A. S., Almuqati N., Ashraf G. A., Hassan N., Qadeer A., Hayat A., Ul Haq M., Hussain I., Murtaza A. (2024). Fuel.

[cit84] Benettayeb A., Haddou B. (2023). Int. J. Environ. Anal. Chem..

[cit85] Mateus G. A. P., Paludo M. P., dos Santos T. R. T., Silva M. F., Nishi L., Fagundes-Klen M. R., Gomes R. G., Bergamasco R. (2018). J. Environ. Chem. Eng..

[cit86] Belbali A., Benghalem A., Gouttal K., Taleb S. (2023). Int. J. Environ. Anal. Chem..

[cit87] Okoya A. A., Olaiya O. O., Akinyele A. B., Ochor N. O. (2020). J. Chem..

[cit88] Sağ Y., Akçael B., Kutsal T. (2002). Sep. Sci. Technol..

[cit89] Mattuschka B., Straube G. (1993). J. Chem. Technol. Biotechnol..

[cit90] Matouq M., Jildeh N., Qtaishat M., Hindiyeh M., Al Syouf M. Q. (2015). J. Environ. Chem. Eng..

