# Supplementary material for: Sustainable wet-spun cellulose-Moringa oleifera composite fibres for potential water purification
Source: RSC Adv. 2025 May 28;15(22):17730–45. doi: 10.1039/d5ra02386f (PMC12117364; doi:10.1039/d5ra02386f)
Supplement: RA-015-D5RA02386F-s001 [file RA-015-D5RA02386F-s001.pdf]

Supplementary Information (SI) for RSC Advances.
This journal is © The Royal Society of Chemistry 2025

Sustainable wet-spun cellulose-*Moringa oleifera* composite filaments for emerging water purification

Abimbola Oluwatayo Orisawayi [a, b*], Prithivi Boylla [a], Krzysztof K. Koziol [a], Sameer S. Rahatekar [a*]

[a] Composites and Advanced Materials Centre, Faculty of Engineering and Applied Sciences, Cranfield University, College Road, Cranfield, Bedfordshire, MK43 0AL, United Kingdom.

[b] Department of Mechanical Engineering, School of Engineering and Engineering Technology, Olusegun Agagu University of Science and Technology (OAUSTECH), Km. 6, Okitipupa-Igbokoda Road, Okitipupa, Ondo State, Nigeria.

*Email: abimbola.orisawayi@cranfield.ac.uk; ao.orisawayi@oaustech.edu.ng

*Email: S.S.Rahatekar@cranfield.ac.uk

Supplementary Material

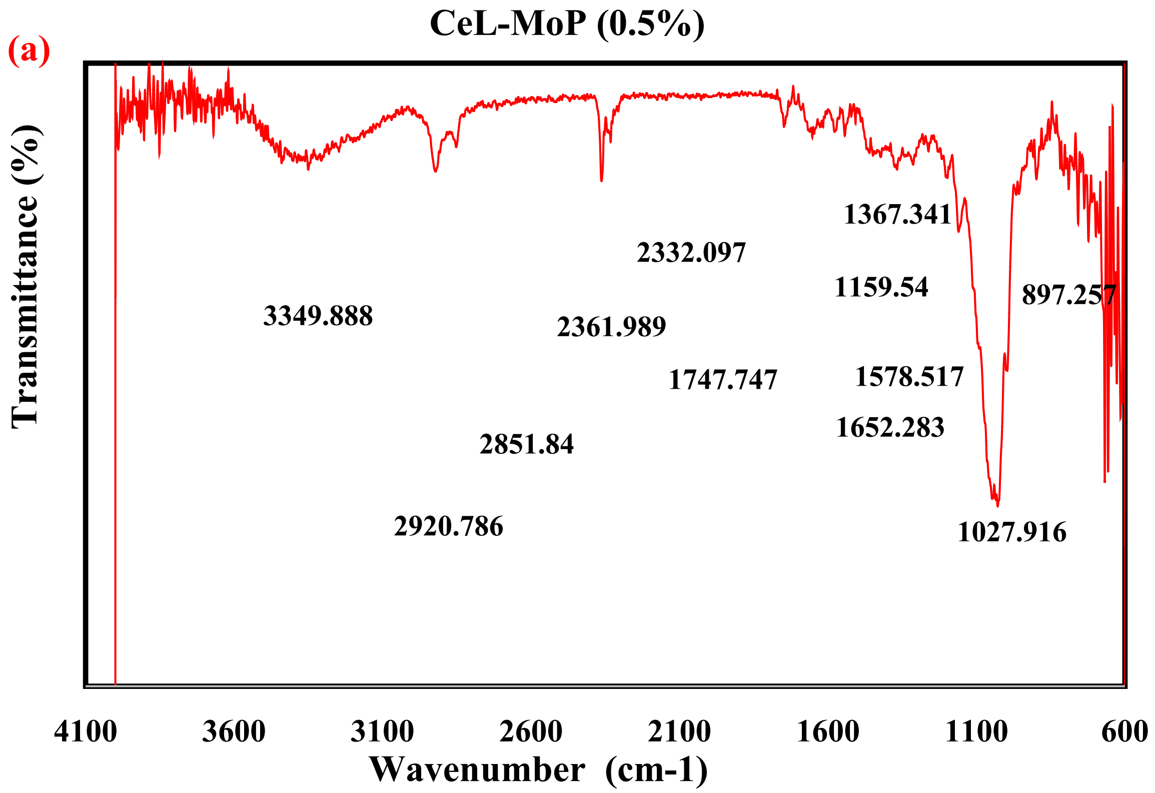

(b)

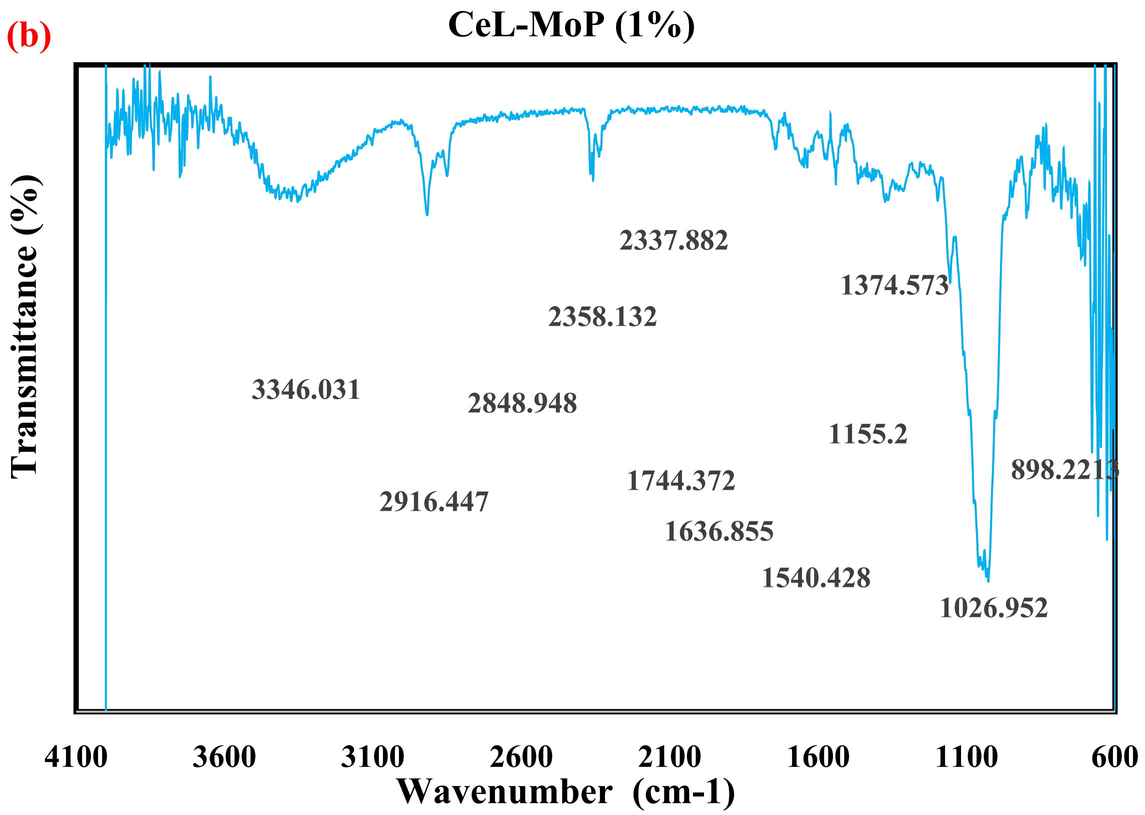

(c)

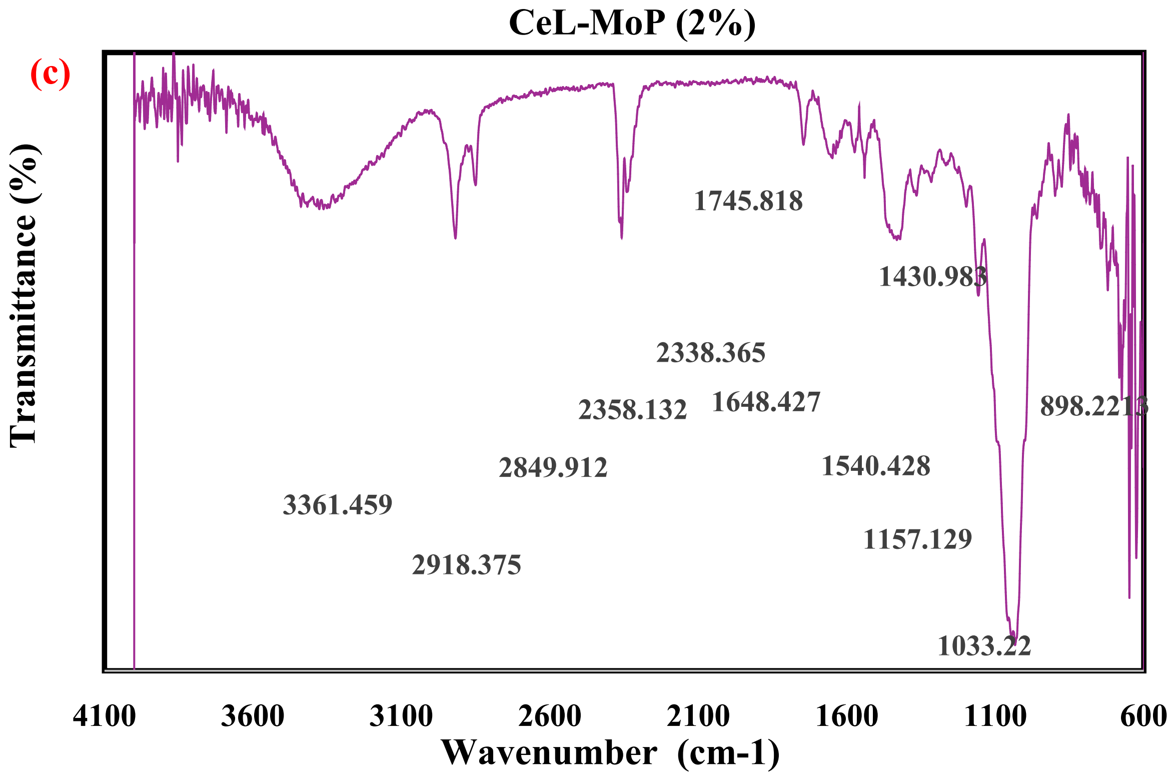

Figure S1 . FTIR spectra of the composite fibres (a) CeL-MoF-0.5%; (b) CeL-MoF-1%; and (c) CeL-MoF-2% before adsorption.

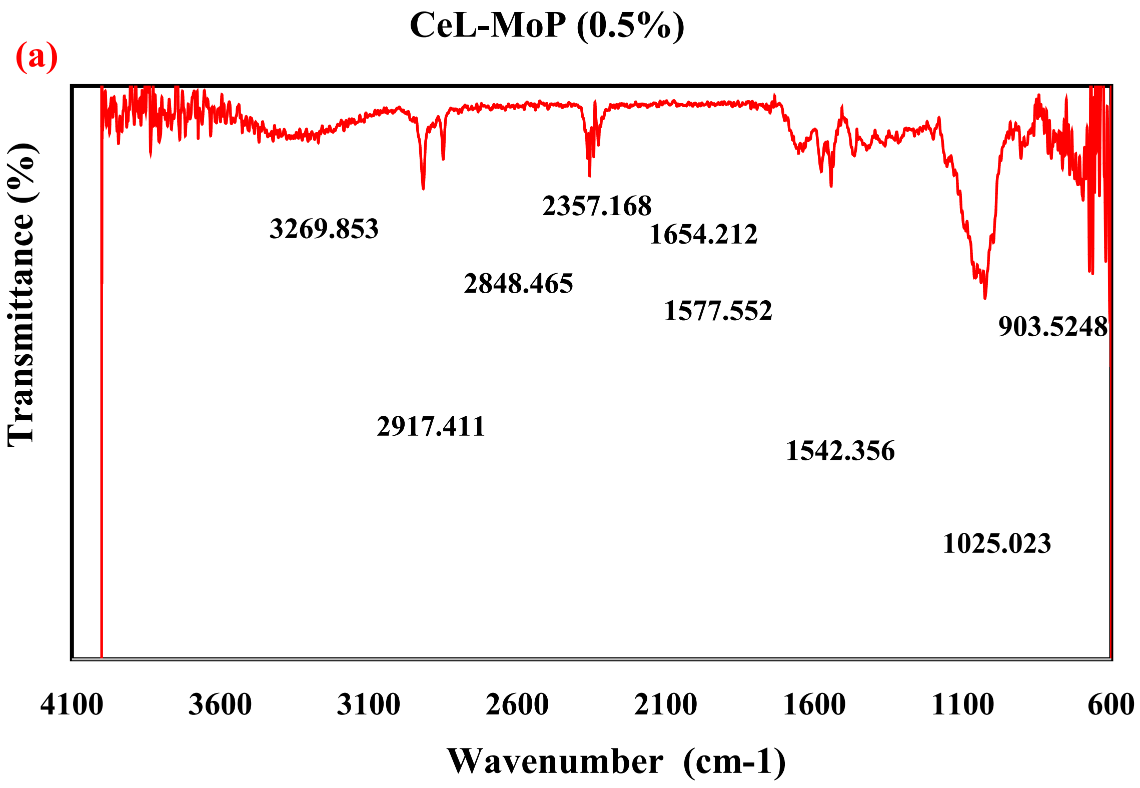
CeL-MoP (0.5%)
(a)
Transmittance (%)
3269.853
2357.168
1654.212
2848.465
1577.552
903.5248
2917.411
1542.356
1025.023
4100
3600
3100
2600
2100
1600
1100
600
Wavenumber (cm-1)

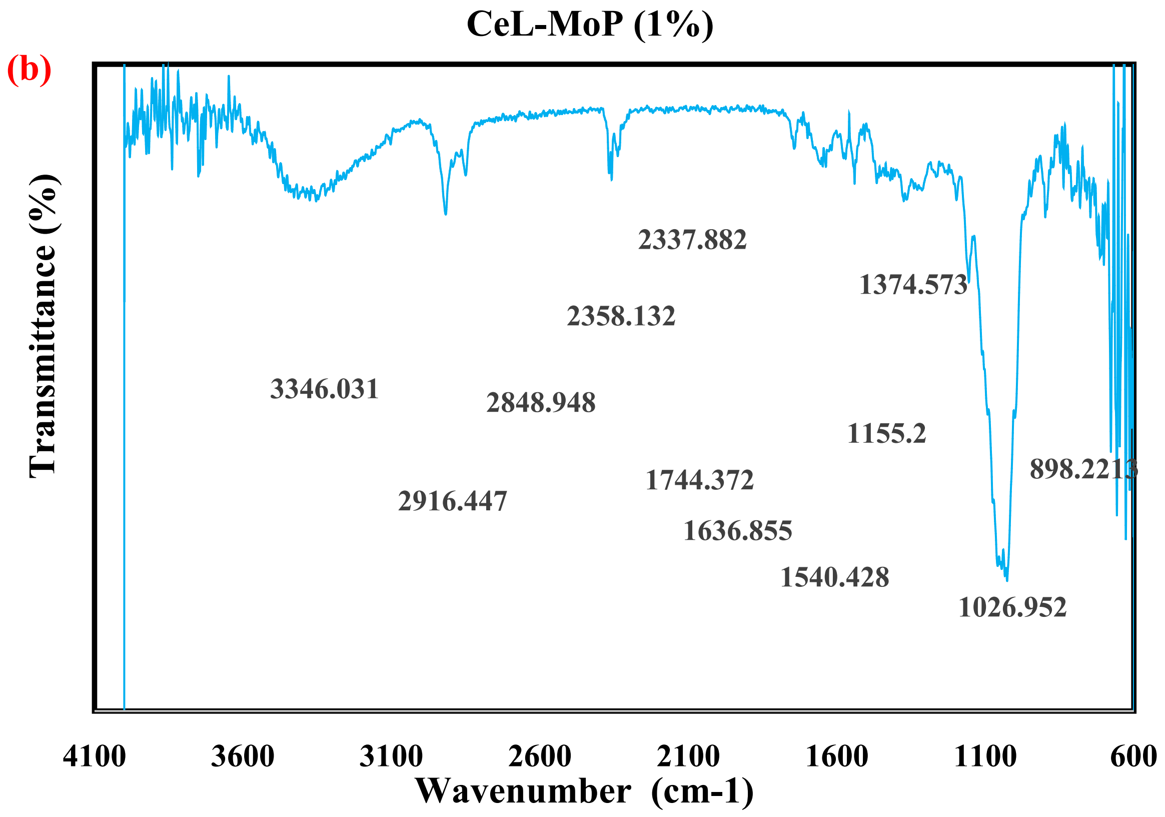
CeL-MoP (1%)
(b)
Transmittance (%)
2337.882
1374.573
2358.132
3346.031
2848.948
1155.2
898.2213
1744.372
2916.447
1636.855
1540.428
1026.952
4100
3600
3100
2600
2100
1600
1100
600
Wavenumber (cm-1)

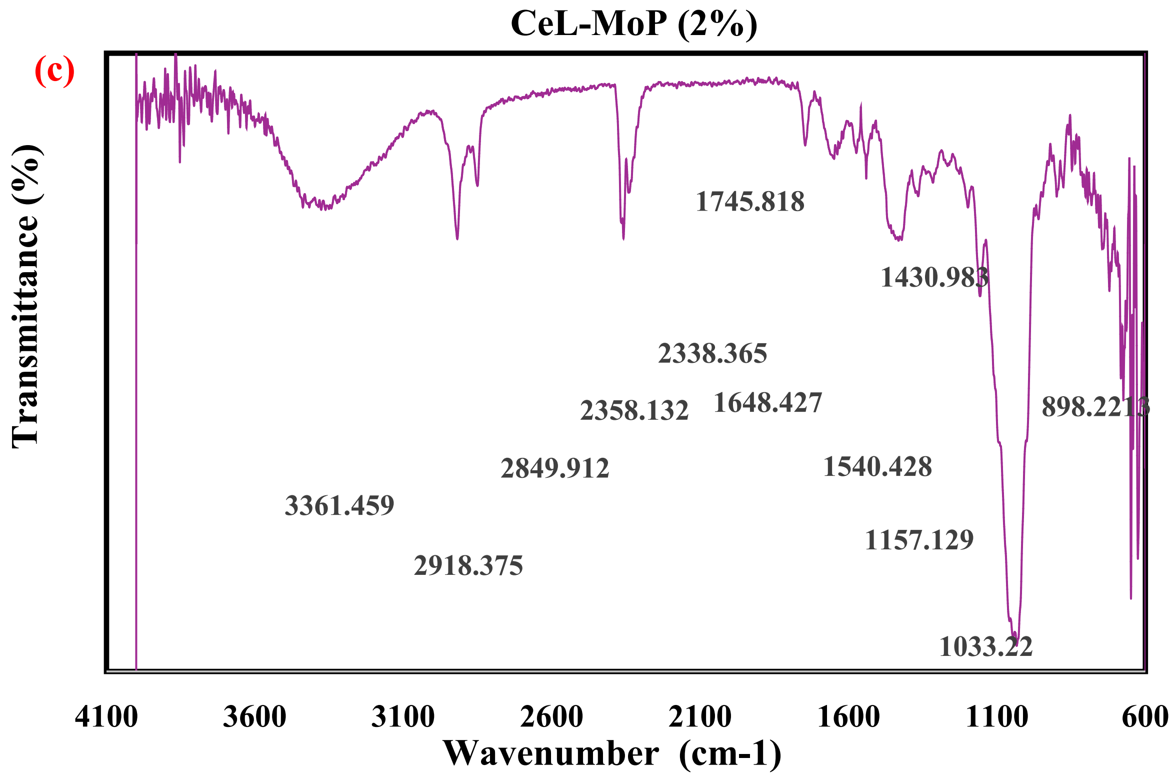

Figure S2 . FTIR spectra of the composite fibres (a) CeL-MoF-0.5%; (b) CeL-MoF-1%; and (c) CeL-MoF-2% after adsorption of Cu^{2+}, Cd^{2+}, and Ni^{2+} .
